# Single-Molecule Fingerprinting of Unlabeled Full-Length Proteins Using an Aerolysin Nanopore

**DOI:** 10.1021/jacs.6c01018

**Published:** 2026-06-29

**Authors:** Verena Rukes, Evita Norkute, Georges Barnikol, Jingze Duan, Jiajie Gao, Chan Cao

**Affiliations:** † Department of Inorganic and Analytical Chemistry, Chemistry and Biochemistry, 27212 University of Geneva; 1211 Geneva, Switzerland; ‡ Institute of Bioengineering, School of Life Science, 27218 École Polytechnique Fédéralede Lausanne (EPFL), 1015 Lausanne, Switzerland; § Institute of Chemical Sciences and Engineering, School of Basic Sciences, EPFL, 1015 Lausanne, Switzerland; ∥ Academy for Advanced Interdisciplinary Studies, 12465 Peking University; 100871 Beijing, China

## Abstract

Proteins play essential roles in cellular processes and are involved in numerous diseases, driving the need for efficient proteoform identification. Recent advances have brought nanopore-based protein fingerprinting within reach, potentially expanding the proteomics toolkit in the near future. Among emerging strategies, label-free, full-length analysis via free translocation is the most promising approach for detecting low-abundance proteoforms. While free translocation is typically accompanied by low temporal resolution, we demonstrate here that such measurements can enable reliable identification even for natural proteins with high sequence similarity. Combining low pH and guanidinium chloride, we generate a strong electroosmotic flow that enables efficient capture and translocation of unlabeled proteins with an aerolysin nanopore. Using machine learning classifiers, we achieve 80% accuracy in distinguishing seven related proteins, based on distinct and directional fingerprints with high reproducibility. Differences in fingerprints partially reflect the distribution of volume and charges in the protein sequences and may contain additional contributions from the translocation dynamics. Our findings position free-translocation measurements of unfolded proteins as a promising approach to fingerprinting without the need for chemical conjugations or enzymatic digestions. With further development, fingerprint-predictions could allow to infer *de novo* protein sequence information from single-molecule data, offering a powerful tool for proteomics.

## Graphical abstract

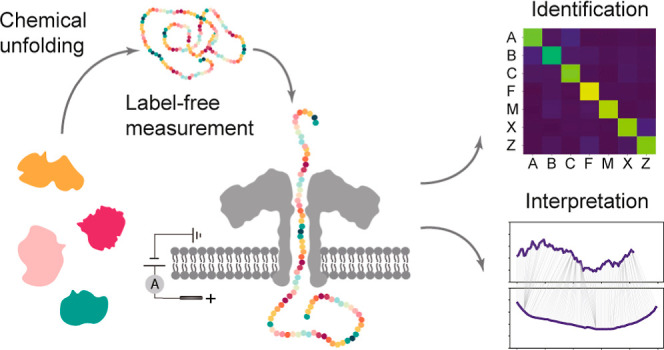

## Introduction

Proteins are the most abundant macromolecules in living systems and fulfill a plethora of functions such as providing cell structure, and signaling pathways, or driving metabolic processes.^1,2^ They are highly diverse, accounting for millions of human proteoforms. Many of these are the product of post transcriptional alterations in the cell that dictate protein function and cannot be accessed through the ∼20,000 protein-encoding genes.^2–4^ This is why tools that directly access the protein level have great significance, e.g. in diagnostics. Currently, protein analysis is mostly based on mass spectrometry (MS), which despite much optimization^5^ is still accompanied by drawbacks such as high costs, limited dynamic range, and low sensitivity as well as short reading length.^6–8^ This has resulted in a rising interest in the development of single-molecule techniques to expand the repertoire of protein-analysis tools.

Recent advancements have highlighted nanopores as promising tools for protein analysis, using voltage-driven ionic currents through a nanometer-scale pore.^9,10^ As analytes interact with the pore, the ionic currents are modulated,^11^ which is commercialized for DNA sequencing.^12^ Notably, nanopores can measure single-molecule interactions and are compatible with mixture samples. To tailor the method to protein analysis, several approaches are being demonstrated, such as using DNA^13–15^ or protein^16,17^ motor enzymes to slowly thread peptides/proteins though nanopores. Another strategy is to fragment proteins and fingerprint the peptide spectra similar to bottom-up MS.^18–20^ However, these require sample processing steps like chemical conjugation or protein digest, that will reduce yield and introduce uncertainty to the analysis.

A direct label-free approach can provide faster detection, specifically of low abundant species, which is desired in protein analysis. This can be achieved by directly measuring the full-length proteins with the pore and identifying them based on prior knowledge of their current readout.^21^ Thereby, unfolding the protein analyte has several conceptual advantages over trying to identify the native proteins. For one, the size range of folded proteins is large, making it difficult to resolve all with one nanopore sensor. Nanopore measurements of folded proteins have great implications when studying protein function. However, information that is buried inside the proteins may be relevant for their identification and is only accessible when the protein is unfolded.

Important groundwork toward measurements of unfolded proteins has demonstrated that chemical denaturants can be used during nanopore measurements to weaken an analyte’s structure.^22^ Since proteins are heterogeneously charged, it is not possible to rely solely on the electrophoretic force (EPF) as a driving force. This has been overcome by taking advantage of the Electroosmotic Flow (EOF), which can attract and transport analytes through nanopores independently of their charge. The EOF relies on the unequal transport of ions species of biological pores (illustrated in Figure 1A), which is referred to as ion selectivity and is caused by the charge of the pore lumen. Using the EOF, proteins and peptides have been successfully translocated through nanopores.^23–25^ Selectivity can be induced and tuned through mutations,^24,26–28^ pH,^18,29,30^ or the electrolyte composition. For instance, guanidinium chloride (GdmCl) has been shown to universally induce EOF in nanopores.^31^

**Figure 1:**
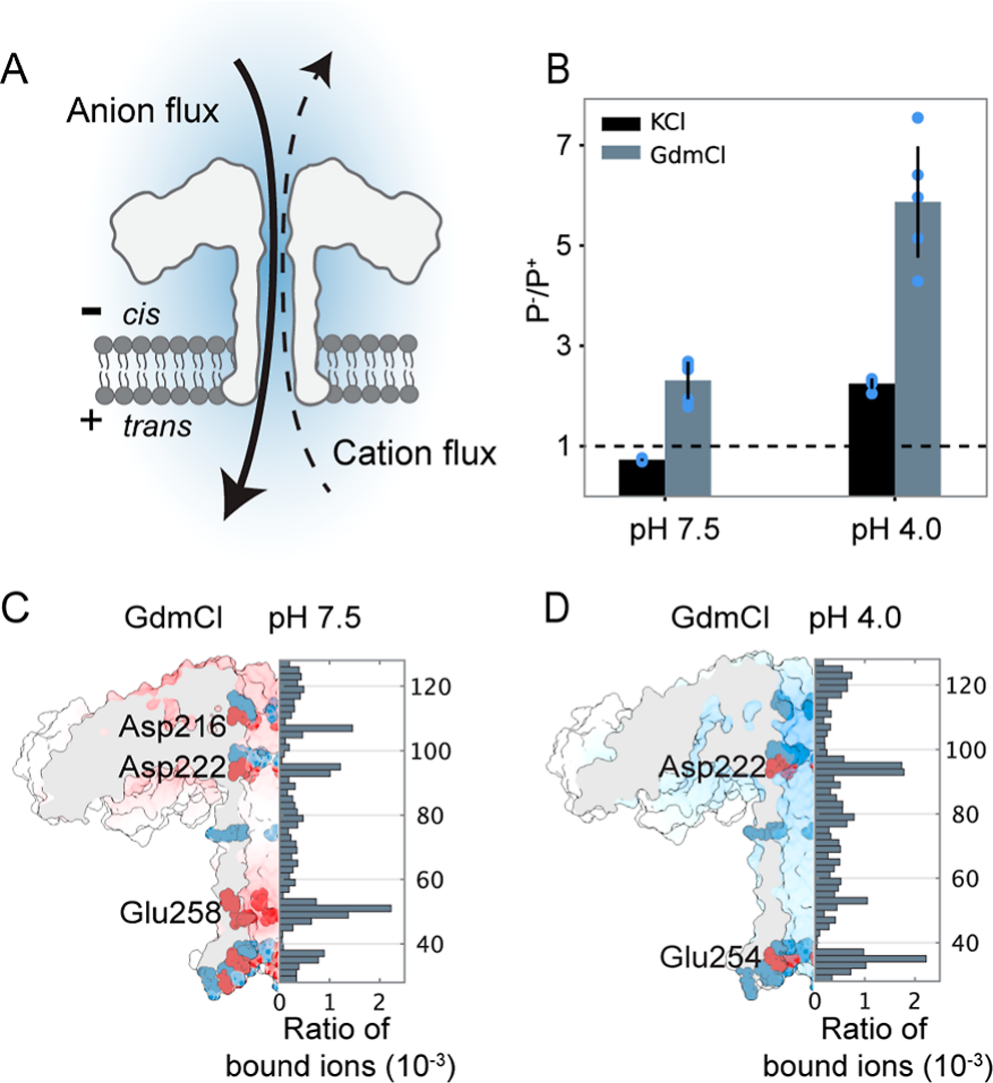
Enhancing ion selectivity by combining pH and GdmCl. (A) Illustration of anion selectivity in aerolysin. (B) Permeability ratios based on reversal potential measurements with aerolysin K238A in KCl or GdmCl at pH 7.5 and pH 4.0. Equal permeability is indicated with a dashed line (*P* ^–^/*P* ^+^ = 1). In each condition *P* ^–^/*P* ^+^ was averaged over >4 independent measurements and standard deviation*s* are indicated. MD results for GdmCl at pH 7.5 (C) and pH 4.0 (D) are shown together with an illustration of the pore structure. Residues carrying negative and positive charge are marked in red and blue, respectively. Bar plots illustrate the duration of Gdm^+^ binding to the pore surface normalized by the simulation time.

Proof of full-length protein translocations through biological nanopores alpha hemolysin (α-HL)^21^ or the homologous pore (CytK)^24^ was recently demonstrated. However, there is so far no estimate of how suitable these systems are for application to protein identification. For instance, there has been no estimate of discrimination accuracy between more than three unfolded proteins at a time. Similarly, it has not been assessed how well proteins with high sequence similarity can be identified against each other. To further develop a nanopore-based method that can benefit from the advantages of label-free identification of full-length unfolded proteins, these key questions need to be addressed. Another essential gap in the development of nanopore tools for the analysis of proteins and beyond, is the lack of understanding how the properties of chemically diverse analytes influence the current readout. To improve the sensing technology, an understanding of its underlying principles is essential.

In this work, we aim to bridge these gaps and expand the understanding of protein analysis with nanopores, by applying the aerolysin nanopore to identification of full-length proteins. Aerolysin has been described as an ideal candidate for linear protein transport.^32^ Other established nanopores are funnel shaped, such as *Mycobacterium smegmatis* porin A (MspA), or contain cavities (such as α-HL), which seems to allow the analyte to assume conformations inside the pore that influence the signal output.^32,33^ On the contrary, aerolysin has a narrow cylindrical shape, which does not allow the formation of substructures inside the pore. Additionally, single-file translocations of an unfolded protein have been reported with aerolysin,^34^ and aerolysin is generally known for its high resolution when distinguishing analytes in free translocation.^35–43^ Specifically, the aerolysin mutant K238A has been used for identification of site specific PTMs in peptides and direct decoding of data-storage polymers.^44,45^ However, to our knowledge, there has been no experimental and analytical investigation focused on establishing this nanopore for identification of full-length proteins.

We demonstrate the optimization of EOF and unfolding conditions in aerolysin for efficient translocation of full-length proteins. In an extensive condition analysis, we discovered that two established parameters that influence the EOF (GdmCl and low pH) can be combined, presenting a simple strategy to generate large EOF without the need for mutagenesis. We then measured 7 proteins from the same Turandot family of *Drosophila melanogaster* in an optimized condition of 3 M GdmCl at pH 4.0, to explore the capability of the nanopore system to discriminate similar analytes. At an optimal potential of 170 mV, machine learning classifiers can discriminate between these 7 analytes with 80% accuracy demonstrating that indeed even freely translocating, similar proteins can be differentiated with nanopores. We further investigated the dependence of the current output on the sequence of the analyte. A simplified biophysical model demonstrated that patterns in the current readout can be partially explained by a synergism of volume exclusion and patterns of positive charges along the protein analyte sequences. Establishing such rational connection is the first step to develop an accurate model, which could significantly enhance this approach and potentially enable *de novo* protein identification.

## Results

### Enhancing EOF in the Aerolysin System

In recent years the central importance of EOF to nanopore protein analysis has been established. When relying only on the EPF many proteins may not be efficiently driven to the nanopore, due to their heterogeneous charge resulting in long durations between single molecule measurements or even no measurement. Contrary to electrophoresis, which relies on the charges of the analyte, electroosmosis relies on the charges of the nanopore lumen and can therefore circumvent the dependence of capture and translocation on the analyte properties. An increasing number of studies show the importance of using EOF to measure peptides and proteins and overcome a dependence on their charges.^24,29,31,46^

We thus characterized the impact of changes in the measurement condition to optimize the EOF in aerolysin K238A. While the structural integrity of the aerolysin pore is maintained up to 3 M GdmCl,^47^ it is known that the EOF in nanopores is affected by both Gdm^+^ ^21,31^ and changes in pH.^29^ Based on reversal potential measurements we calculated the ratio of permeability (*P* ^–^/*P* ^+^) between anions and cations through the pore (Figure 1B and SI Figure S1). This ratio is commonly used to determine the direction and relative magnitude of ion selectivity in biological nanopore systems.^24,27,31,48,49^ Aerolysin K238A is slightly cation selective at neutral pH in KCl (*P* ^–^/*P* ^+^ = 0.73 ± 0.02). By changing the pH to 4.0 or changing the electrolyte to GdmCl, we observed a strong anion selectivity, resulting in *P* ^–^/*P* ^+^ = 2.24 ± 0.10 and *P* ^–^/*P* ^+^ = 2.31 ± 0.37, respectively. When combining both conditions (GdmCl at pH 4.0), an even larger selectivity is reached with *P* ^–^/*P* ^+^ = 5.87 ± 1.11 in aerolysin K238A. Thus, we establish the combination of low pH and GdmCl ions as a fast and simple strategy to turn a slightly cation selective pore system into a strongly anion selective one.

The increase in EOF by combining Gdm^+^ and low pH is surprising, since the more positively charged pore lumen at low pH could negatively interfere with Gdm^+^ binding. To investigate how or whether the binding of Gdm^+^ is impacted by the change in pH, we have performed all atoms molecular dynamics (MD) simulations with aerolysin K238A in KCl and GdmCl both at pH 7.5 and pH 4.0. Interaction spots of the ions with the pore were defined, by considering ions that enter the pore and monitoring the time that each ion type spends in close proximity along the surface of the pore lumen, similar as previously reported.^31^ We then normalize by the duration of the trajectory (see details in Supporting Information methods). Gdm^+^ showed specific interaction spots along the pore at both pH. At pH 7.5, Gdm^+^ lingered at residues Asp216, Asp222, and (most prominently) Glu258, resulting in distinct peaks seen in Figure 1C. At pH 4.0, the peaks at residues Asp216 and Glu258 largely disappear. While Asp222 remained a site of interaction, the residence of Gdm^+^ at Glu254 increased (Figure 1D). At either pH such peaks did not occur for Cl^–^ in the GdmCl condition or for the ions in a KCl condition (SI Figure S2). We additionally estimated the EOF in our simulated systems as the net transport of water molecules through the pore over time (SI Figure S3). This yielded fluxes from *cis* to *trans* of 0.922 water molecules/ns in GdmCl at pH 7.5 and 2.022 water molecules/ns in GdmCl at pH 4.0. This 2.2-fold increase in simulated EOF is comparable to the experimentally assessed effect of the pH shift on the permeability ratio in GdmCl, which increased 2.5-fold.

Overall, MD results—in agreement with experiments—showed that Gdm^+^ ions can bind the pore lumen both at pH 7.5, and at pH 4.0. At both pH, the Gdm^+^ interaction spots align well with negative charges in the lumen, showing the importance of electrostatic interactions for the binding. The combination of low pH and GdmCl increases the *P* ^–^/*P* ^+^ by more than 2-fold without noticeable negative interference, therefore providing a simple strategy to produce extreme ion selectivity and EOF.

### Optimizing the Measurement Condition for Protein Translocation

To optimize the buffer condition for full length protein analysis with aerolysin K238A we used Turandot A (TotA) from *D. melanogaster* as an analyte. At pH 7.5, TotA has a net charge of −7.8 and is driven toward the pore by both the EPF and the EOF, while at pH 4.0 the protein’s net charge is +8.0 and therefore the EOF is the only attracting force, opposing the EPF (Figure 2A). The TotA protein has 115 amino acids (aa), and its native structure is composed of four α-helices.^50^ This is reflected by the Circular Dichroism (CD) spectrum we measured in water, which yielded 100% helix content (Figure 2B). CD measurements further showed that TotA is partially unfolded at 2 M GdmCl and largely unfolded at 3 M GdmCl. In contrast, lowering the pH from 7.5 to 4.0 had no effect on the CD spectrum of TotA, indicating that its secondary structure remains unchanged under these conditions (Figure 2B). Therefore, by measuring the protein at 2 M GdmCl and 3 M GdmCl both at pH 7.5 and pH 4.0, we investigate the importance of residual structure, and the EPF-EOF interplay in aerolysin measurements of a full-length protein. TotA interactions with the aerolysin K238A pore were measured under a voltage range from 100 mV to 210 mV applied to the *trans* compartment. In all conditions and at all voltages, the analyte was added to the *cis* compartment, which caused deep current blockages with less than 15% mean residual current and dwell times with distribution peaks between 3 and 30 ms (Figure 2C–E, SI Figure S4).

**Figure 2:**
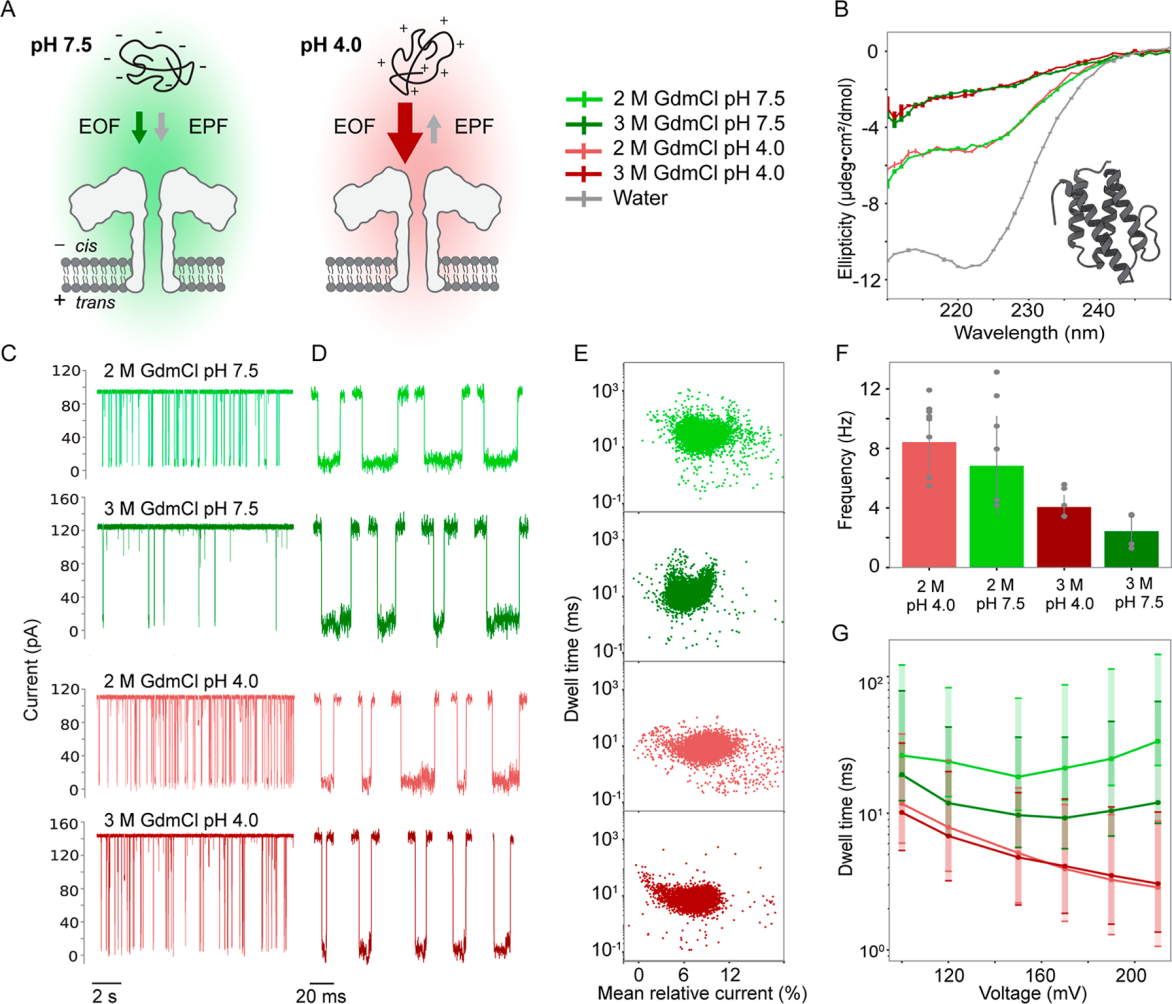
Optimizing conditions for protein translocations. (A) Illustration of the interplay of EOF and EPF in GdmCl at pH 7.5 (left) and pH 4.0 (right). (B) Results of CD measurements are averaged over 3 repeated measurements; the standard deviations are indicated. The TotA structure is shown (PDB: 8PBV). (C) 10 s of raw trace for each condition filtered to 1 kHz for illustration. (D) Example events filtered to 10 kHz for illustration. (E) Dwell time versus current scatterplots for individual events and each condition recorded at 120 mV. The event numbers are listed in SI Table S2. (F) Frequency of events calculated by fitting the interevent time of individual pores. Averaged over 8, 6, 5, and 4 recordings from left to right respectively weighted by the number of events in each recording, standard deviations are indicated. (G) The distribution of log dwell times for each condition at each voltage were fitted using a single exponentially modified Gaussian distribution. The peak and the half width of the fitted distributions are shown in a semilogarithmic plot.

We found that generating a strong EOF even against the EPF (pH 4.0) is the more efficient strategy to capture and translocate the protein analyte, compared to a combination of EPF and lower EOF (pH 7.5). As shown in Figure 2F, at 100 mV shifting the pH from 7.5 to 4.0 increased capture at both 2 and 3 M GdmCl, confirming that there is a stronger driving force toward the pore at low pH, even against the EPF. Strikingly, at pH 7.5, the fitted dwell times did not decrease monotonously with increasing voltages (Figure 2G). We observed a turnover voltage of 150 mV in 2 M GdmCl, which shifted to 170 mV at 3 M GdmCl. Translocation can be viewed as an energy barrier crossing event, where the net driving force produced by the electric field lowers the effective barrier height, resulting in typically exponential decrease of event duration with increased applied potential.^51^ Consequently, a continuous decrease in dwell times with increased potential bias is commonly used as proof of translocation in the nanopore field.^21,24,52^ Durations of other interactions like docking can show no or inverse dependence on the applied potential. Therefore, we cannot conclude that TotA translocates the pore at pH 7.5. However, despite being captured against the EPF, the analyte did translocate the engineered aerolysin pore at pH 4.0 in both 2 and 3 M GdmCl as confirmed by the decrease in dwell times with increasing voltage (Figure 2G, SI Figure S5). Interestingly, the residual structure of TotA in 2 M GdmCl did not hinder its translocation. At pH 4.0, the current pattern of the events as well as the shape of the event populations was very similar in 2 and 3 M GdmCl, further indicating that the change in structure of TotA between 2 and 3 M GdmCl did not impact the process of its translocation.

Further, our data indicates that the influence of GdmCl concentration on the driving forces that acted on TotA is highly complex. Increasing the GdmCl concentration from 2 to 3 M increased the open pore current at both pH (SI Figure S6). However, while dwell times were shorter at pH 7.5 with increased salt concentration, they remained similar at pH 4.0. We also observed that the capture frequency decreased with increasing GdmCl concentration. This points out important differences between our system and previous observations with wt-aerolysin in KCl at neutral pH.^25^ In this slightly anion selective system, increasing the salt concentration from 1 to 4 M KCl enhanced the capture of peptides, which led to the speculation that a larger total ion flux can promote a more potent EOF. In contrast, the decreased capture frequency observed at 3 M GdmCl in our system could arise from an increased binding of Gdm^+^ to the protein analyte in this condition. Higher GdmCl concentration may modify the effective analyte charge through ion association and charge screening, making the analyte appear more positively charged and thereby lowering the capture frequency.

### Measurements of a Protein Family

The development of nanopore-based protein fingerprinting systems is rapidly progressing. While strategies to transport proteins have been focused on, there is little understanding of what protein features lead to nanopore discrimination or what the limitations of this approach are. These are, however, key questions to address for the continued development and future optimization of nanopore-based protein analysis. We decided to explore this gap by measuring an array of very similar proteins in the optimized condition (in 3 M GdmCl, pH 4.0). We chose seven members of the Turandot family of proteins, which have large structural similarities and pairwise sequence identities between 38% - 70% (Figure 3A, SI Figure S7). These analytes are ideal targets since they are natural proteins with no destabilizing mutations or labels and tags that could influence their measurement.

**Figure 3:**
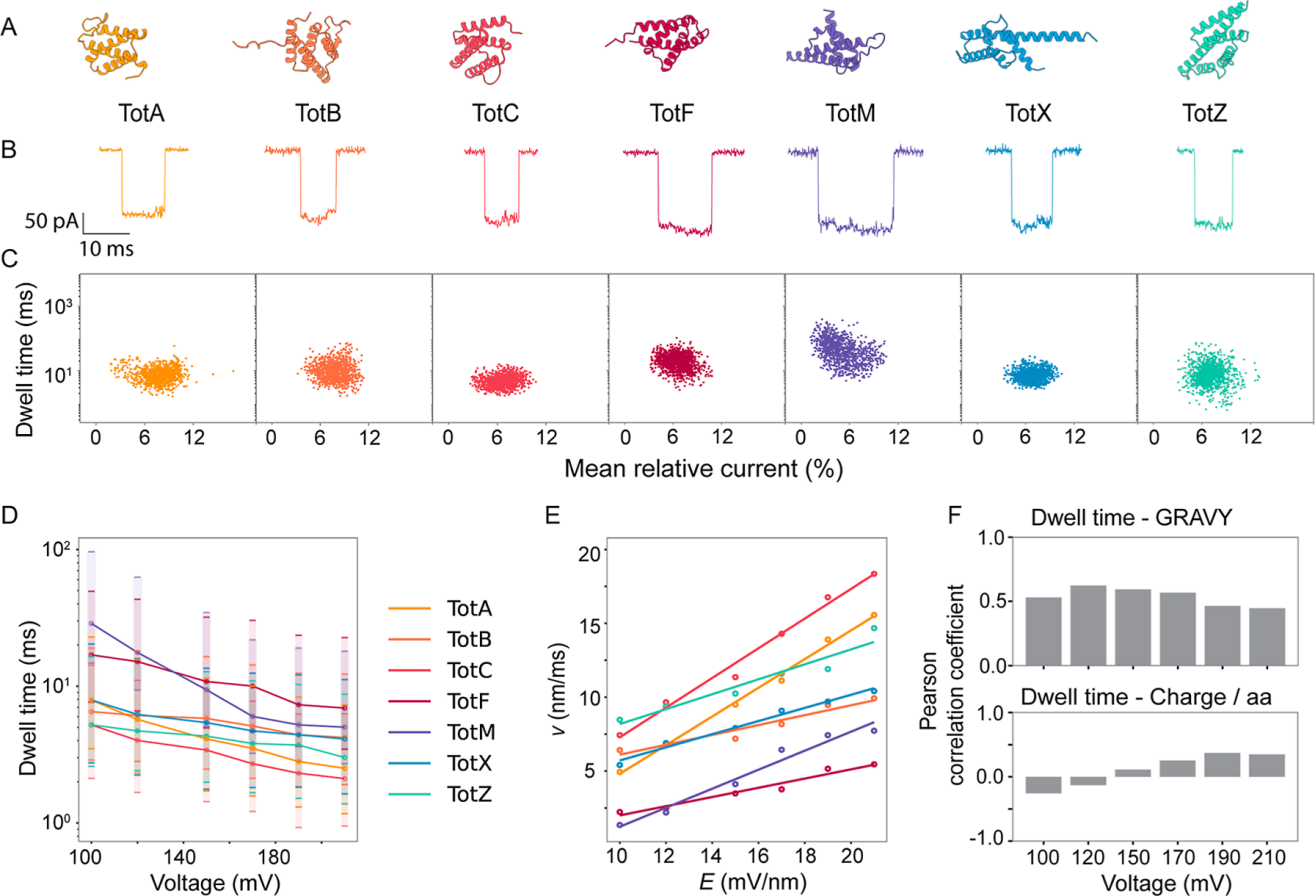
Measurements of related proteins. (A) Structural representation of each protein analyte (predicted using Alpha Fold 2).^53^ (B) Example events collected at 120 mV for corresponding proteins. (C) Dwell time versus current scatterplots for individual events recorded at 120 mV of (left to right) TotA (yellow), TotB (orange), TotC (light red), TotF (dark red), TotM (purple), TotX (blue), TotZ (turquoise). Event numbers are listed in SI Table S4. (D) Population dwell times were fitted with the Fokker–Planck equation for each protein and plotted against applied voltages. Maximum and halfwidth of the distributions are shown. (E) Velocities of each protein against electric field fitted with a linear regression. Regression results in SI Table S5. (F) Pearson correlations between the population dwell time and the GRAVY (top) and the charge normalized by the number of amino acids of each protein (bottom) cross different voltages. The *p*-values in SI Table S6.

The analytes produced event populations with 0–12% mean residual current at 120 mV and clear deviations in dwell time between the different proteins (Figure 3B,C, Example raw traces in SI Figures S9–S11). For all 7 proteins, the population dwell time decreased with increasing voltage, confirming their translocation (Figure 3D, SI Figures S12–S14).

Translocation velocities (Figure 3E, proteins length divided by the fitted dwell time) showed a roughly linear dependence on the applied electric field, which is in alignment with expectations (SI Note 2). However, velocities are not consistent between analytes, with the dwell times poorly correlating with the analyte length (SI Figure S15). This suggests that sequence-specific effects dominate the kinetics during protein translocation. The grand average hydropathy (GRAVY, calculated based on the Kyte-Doolittle hydropathy scale^54^) showed a weak positive correlation with the population dwell time across all voltages (Figure 3F), while the charge per amino acid (aa) did not consistently correlate with the dwell times. Thus, while the velocity depended linearly on the electric field, the sequence of the analytes affected the absolute dwell times in a nontrivial manner.

### Machine Learning-Based Protein Identification

Nanopore protein identification ultimately has the potential to identify even low abundant molecules in undefined samples. This requires the development of advanced analytical methods capable of identifying proteins through their single-molecule interactions. Machine learning classifiers have demonstrated strong performance when tackling this challenge.^16,21,55^ Since to our knowledge there has been no discrimination of several highly similar unfolded full-length proteins, we found it important to not only determine how well the analytes can be discriminated, but also what the basis of discrimination was. We aim to address whether a measurement system this simple—with unlabeled proteins in free translocation—can capture enough analyte information in single molecule events to be transformed into an identification platform.

We therefore chose to describe each single-molecule interaction in 88 features (Figure 4A, SI Table S8) for an interpretable analysis. While some features described the whole interaction (such as dwell time, mean current, mean of the absolute gradient) others were calculated on segmented events^21^—allowing us to include progressive developments along the length of the events. Like others previously^55^ we also extracted features from the power spectrum. Additionally, we added specific features tailored to our analysis: for instance, to grasp current substates of the events, current from each event was clustered into two parts and the mean of each was extracted.

**Figure 4:**
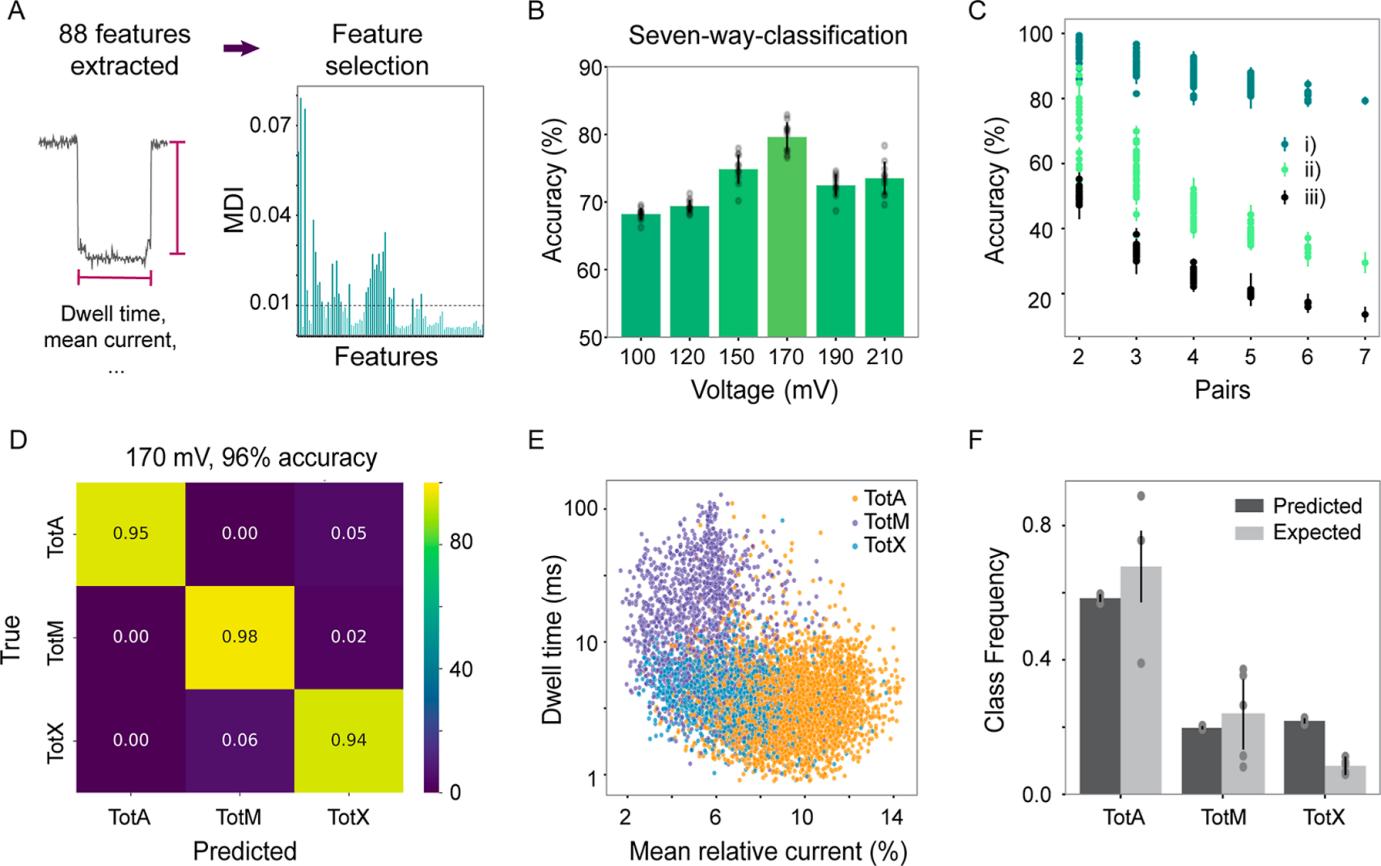
Machine learning based protein identification. (A) Illustration of the feature extraction and reduction based on the mean decrease in impurity (MDI) for machine learning classification. (B) Accuracy of seven-way classifications of the Tot family proteins using data at different voltages. For each voltage 10 rounds of data selection and model training were performed and the average with indicated standard deviation is shown. Event numbers are listed in SI Table S7. (C) Classification accuracies for all possible combinations of Tot proteins in 2 to 7-way classifications using data at 170 mV. The performance of three approaches is compared: i) main model with the relevant out of 88 features (same as panel B), ii) same approach but using only dwell time and average residual current as features, iii) dummy classifier randomly guessing the protein identity. For each combination of proteins three rounds of data selection and model training were performed and the average with indicated standard deviation is shown. (D) Confusion matrix for the 3-way classification of TotA, TotM and TotX. (E) The scatterplot of mixture data colored by predicted label (3 M GdmCl pH 4.0, 170 mV). (F) Comparison of expected and predicted relative abundance of labels in the mixture data. The prediction values are averaged over three models trained on different randomly selected events with standard deviation indicated. The expected values are averaged over frequency results from different recordings and error bars represent the standard deviation of this average.

To assess the utility of all extracted features the mean decrease in impurity (MDI) in a random forest classification was used (SI Figure S16), where larger values indicate a higher utility. One of the most important features was the dwell time, showing that the protein-dependent variance in dwell time can ultimately be useful for identification. On the contrary, other features contributed very little to the classification. For instance, the skewness and mode of the 8 equal event segments showed a small MDI. For efficiency, we selected features with an MDI above a threshold of 0.01. We then benchmarked common classification algorithms and finally trained a voting classifier combining the top three models (see Supporting Information methods).

Interestingly, we found that tuning the applied voltage affected classification accuracies (Figure 4B). For instance, at 120 mV an accuracy of 69 ± 1% was achieved, while increasing the voltage to 170 mV improved accuracy to 80 ± 2%. However, further increase in voltage (>170 mV) reduced accuracy, potentially due to the shorter event times at those voltages. This suggests that the voltage differently affected the analytes interactions with the pore leading to an optimal voltage for differentiability at 170 mV. This demonstrates a so far underappreciated effect of the voltage on discrimination in nanopore measurements.

These accuracies show that nanopore protein fingerprinting can distinguish even closely related and unlabeled proteins. To explore the impact of increasing the number of proteins on discrimination accuracies, we trained models on all possible combinations of proteins in 2–7-way classifications (Figure 4D). We compare (i) the main model with the relevant features out of 88 dimensions, to (ii) models that are only trained on the features dwell time and current, and (iii) dummy classifiers that represent a random guess of the protein identity. Accuracies with the dummy classifiers decayed fast with more proteins added, demonstrating the increasing challenge of randomly guessing a proteins identity correctly from a larger pool. When using only the features dwell time and current, accuracies consistently outperformed the random guess, even reaching >80% accuracies for some pairwise classifications. However, increasing the number of proteins rapidly decreases accuracies, since there is substantial overlap in dwell time and current between the proteins. Using the added statistical features in our main models helped to maintain high accuracies for all combinations of proteins. This shows that in the progressive changes along the events (that go beyond an average of the whole interaction) enough information is captured even from these relatively quick interactions between analyte and nanopore.

We further tested one of the classifiers in a mixture experiment. Three separate classifiers were trained on data corresponding to analytes TotA, TotM, and TotX, achieving an accuracy of 95–97% on a labeled test set (with one of the confusion matrices shown in Figure 4D). In the mixture experiment, data was collected while all three proteins were present simultaneously, and the classifiers were applied to predict the identities of the molecular interactions (Figure 4E). Since it is not possible to determine the true analyte responsible for each interaction in a mixed environment, we compared the predicted class frequencies instead. These frequencies indicate the proportion of events assigned to each class by the models. As a reference calculated expected values based on interaction frequencies in measurements obtained from separate individual-analyte samples. As shown in Figure 4F, the predicted class frequencies closely match the expected ones, with TotA contributing a higher number of signals than TotM or TotX.

Overall, we demonstrate a simple approach with the advantage of measuring unlabeled full-length proteins. Measuring full-length proteins means that all analytes block a large portion of the current, making it difficult to distinguish based on current blockage alone which is possible with some short analytes.^42,56^ However, our machine learning-based analysis demonstrates that adding more dimensions can help to overcome this hurdle and lead to impressive accuracy.

### Interpreting Nanopore Current Based on Protein Sequences

As described above, discrimination of the related proteins in our system relies on a combination of different features including dwell time, current states along the signals, standard deviations and gradients. A hurdle in the development of nanopore technology is the limited understanding of how features of the analyte exactly lead to the observed current output. To advance the understanding of signal-sequence interplay, we thoroughly analyze the current signature of each analyte and explore how well sequence features can account for them.

For an unbiased display of current states, we randomly selected events stemming from each analyte and overlaid them in a single plot for detailed comparison (Figure 5A, SI Figure S17). We found that the current signature of each analyte is directional, which is most evident in the patterns for TotF, TotM, and TotX. This likely contributed to the discrimination of the analytes, as it increased uniformity in the signals. For TotA, TotB and TotC, the currents tended to slightly decrease around the middle of the event, while for TotX an increase in current appeared toward the middle of the interaction. TotF and TotM showed a clear downward trend in current and TotZ had the most stable current. Unidirectionality of signals were previously reported in measurements of full-length proteins with α-HL^21^ when the analyte was tagged with a negatively charged peptide to force directional entry. A plausible explanation for our observation of directional signals is thus, that our system achieved directional capture without the need for a tag.

**Figure 5:**
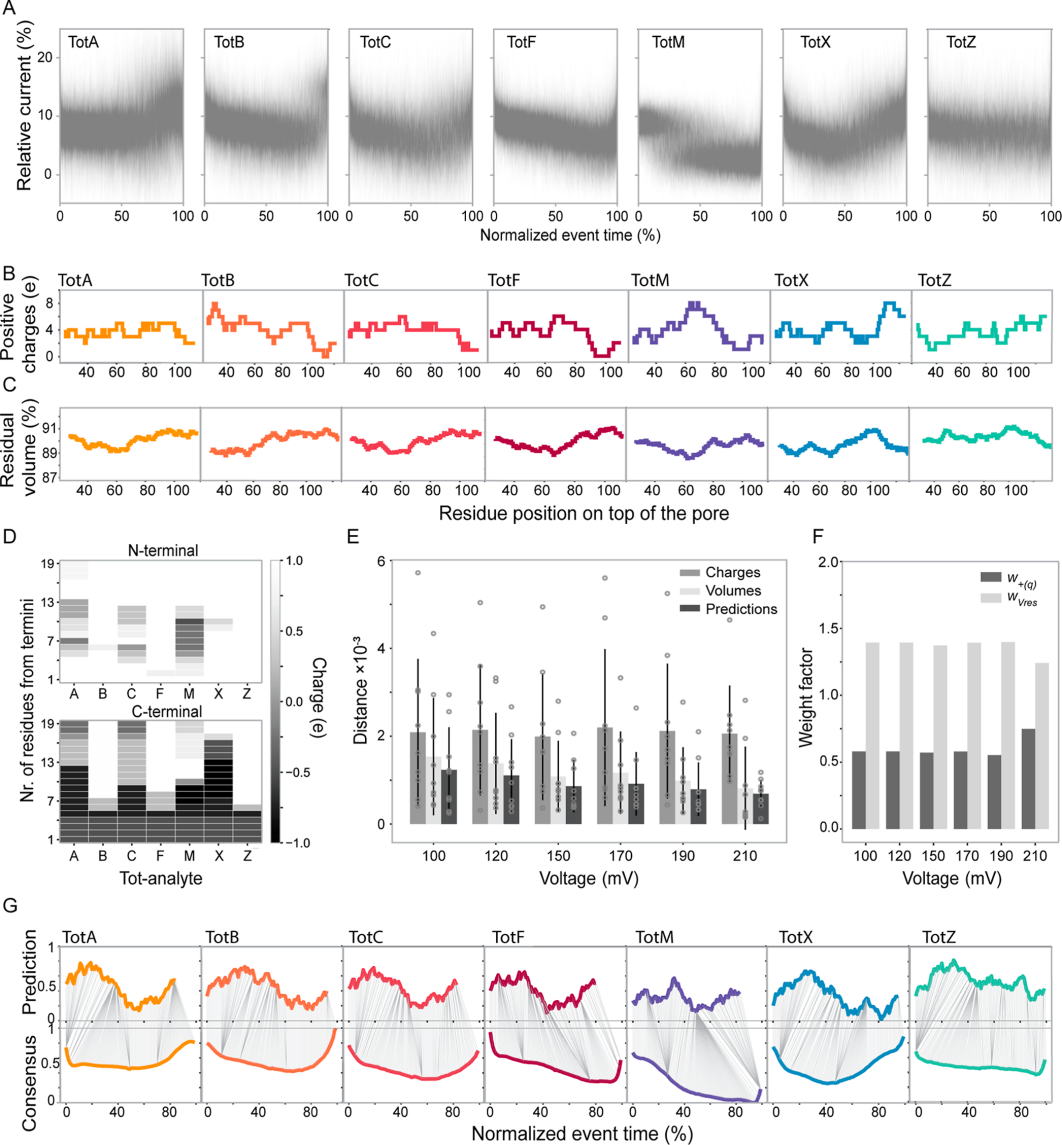
Interpretation of current signals. (A) Time normalized overlay plots of 800 randomly chosen events for each Tot family protein recorded at 100 mV. Sum of positive charges (B) and relative residual volume (C) for a sliding window of 30 amino acids along the sequence of TotA (yellow), TotB (orange), TotC (light red), TotF (dark red), TotM (purple), TotX (blue), TotZ (turquoise). Overlay plots of each protein are shown in SI Figure S17. (D) Display of terminal charge in a color scale at pH 4.0 when considering a range of 1–19 residues from the N-terminus (top) and C-terminus (bottom) for each Tot family protein. For optimal interpretability the color scale highlights differences between −1 to 1 elementary charge. (E) Weighted DTW distances between the consensuses and positive charges (dark gray), residual volumes (light gray), and the prediction (black) shown at each voltage. Bars represent the average with values from individual proteins shown as dots. (F) Weight factors for positive charges (dark gray) and residual volumes (light gray) optimized for the predictions for each voltage. (G) DTW alignment paths between the predictions and consensuses for TotA (yellow), TotB (orange), TotC (light red), TotF (dark red), TotM (purple), TotX (blue), TotZ (turquoise) at 100 mV (SI Figures S23 and S24 for all voltages).

To interpret the current signatures based on analyte sequences, we explore a simplified model that represents the physicochemical properties of the analytes along the sequences. We assume that the simultaneous effect of all residues inside the pore lumen equally contribute to one current level and considered a sliding window of size *l* = 30 aa—corresponding to the approximate pore height—gliding along the analytes and monitored the parameters: sum of positive charge (Figure 5B) and residual volume (Figure 5C) in each window (SI Figure S19). Volume exclusion is a typical model to explain current fluctuation in nanopore sensing.^57^ For instance, when analyte-motion through MspA or CsgG was controlled using a Helicase^13,58^ or ClpX^16^—the volume of the amino acids within the reading site often correlated with the observed current blockages. We therefore consider this factor as the relative residual volume during protein translocations as $V_{res}=\frac{\left(V_{pore}-V_{30aa}\right)}{V_{pore}}\times 100\%$ , where *V*_30aa_ is the summed volume of aa in each sliding window (see Supporting Information methods for details). The influence of positive charges was specifically considered, because experiments with designed control analytes showed that positive charges increased the event current, while negative charges had little influence on the event current in this condition (SI note 3, SI Figure S21).

When estimating how well properties along the sequences align with the signature current levels, the orientation of entry into the pore is important. Our analysis suggests that most proteins were captured by the C-terminus indicated by the charges at the termini of the Tot family proteins. Considering different distances from the terminus, the C-terminus showed lower summed charge than the N-terminus for all proteins at most distances (Figure 5D). This is largely due to the p*K*_a_ of the terminal groups giving a full positive charge to the N-terminus at pH 4.0 and a partial negative charge to the C-terminus. Notably, these are theoretical charges while the effective analyte charge will be further affected by screening through interactions with the denaturant electrolyte. We therefore discuss the more likely C-terminal capture here and show the same analysis for N-terminal capture in SI Figure S22.

To estimate the similarity between sequence parameters and signal patterns, we generated consensuses that represent the current signals for each protein analyte at each voltage using Savitzky–Golay filtering (see details in Supporting Information methods). Dynamic Time Warping (DTW) distances were chosen as a measure for similarity between the consensuses and sequence features, due to the possible variability in time spent inside the pore for different parts of the analyte. DTW distance takes this into account and provides a measure for similarity between analyte properties and current signals, with smaller distances signifying a better agreement. We found that on average volume along the sequence showed better agreement with the signals than the charges (Figure 5E). A large spread in distances is found between analytes, which could be caused by different translocation dynamics, meaning that some analytes may be more inclined for stalling or acceleration during translocation than others, resulting in variations between distances. For most proteins the same trends of better agreement with volumes are observed when considering the individual analytes (SI Figure S22D). Further we sought out to provide a more comprehensive model that combines both factors into one prediction model given by

$$prediction=w_{\left(+\right)q}\times \left(+\right)q+w_{V_{res}}\times V_{res}$$

with (+)*q* the sequence of positive charges and *V*_res_ the residual volumes in the sliding windows. Respective weights *w*_(+)*q*_ and *w*_*V*res_ were optimized for minimal distance to the consensuses (see details in Supporting Information methods). Notably, by considering the charges and volumes (which include molecular weight as well as hydropathy^59^) this provides a comprehensive representation of the physicochemical properties of the protein analytes. One prediction was optimized for all measured protein analytes together, since the prediction should be generally applicable. However, since patterns with the same analyte changed with the voltage (SI Figures S17 and S23), a new set of weights was optimized for each measured voltage. As seen in Figure 5F, the weight for the volumes was consistently larger, with a lesser contribution stemming from the charges. The predictions showed on average better agreement with the consensuses across all voltages (Figure 5E, SI Figure S22D). We further illustrate the prediction-consensus warping paths in Figure 5G and SI Figures S23 and S24 for all analytes and voltages.

Overall, we observe that analytes produce quite uniform current states when translocating aerolysin. These states may stem from contributions both from charges and volume exclusion along the signal sequence. However, the variability in the signals is larger than that seen in the predictions that combine those factors. We therefore propose that a large part of the signal differences between the analytes may stem from the translocation dynamics. Since the translocation process of TotA was not affected by the change from 2 to 3 M GdmCl (Figure 2E,G) we do not have an indication that residual structure is the cause of the signal patterns. However, heterogeneity may also arise from additional factors. Variations in charge distribution along the sequence could cause different regions to translocate at distinct rates. Furthermore, specific residue-pore lumen interactions within aerolysin may induce transient stalling events. As a result, the number of residues occupying the pore is unlikely to remain constant throughout translocation, further contributing to signal variability between analytes. These possibilities merit systematic exploration in future studies, for instance through extensive MD simulations.

## Discussion

As the field of nanopore protein analysis evolves, the technology is poised to become indispensable, offering speed, affordability, and portability compared to traditional methods.^60^ In this study, we investigated the capabilities of an aerolysin nanopore for label-free fingerprinting of full-length proteins and advanced the understanding of how nanopore signals and protein sequences depend on each other. Overall, our findings demonstrate the potential of fingerprinting full-length, unfolded proteins in free translocation. This label free approach without sample processing steps like chemical conjugation or enzymatic digests could offer the identification of low abundant species in mixture samples.

Our analysis underscores the critical role of the EOF as a driving force in protein measurements. While the long and narrow barrel of aerolysin provides a big surface that can yield large ion selectivity, it also provides more hydrodynamic resistance compared to other nanopores which reduces the resulting water flux.^31^ This explains why in 2 M GdmCl at pH 7.5, TotA cannot translocate through aerolysin, whereas other proteins have been translocated through α-HL under the same condition.^21^ Here, we overcome this challenge by combining GdmCl with a low pH condition which induces an even larger EOF. We observed that the residual structure of TotA at 2 M GdmCl did not impact its translocation or signal patterns. This is an important advantage, as even at 3 M GdmCl, some protein structures may remain. Generating reliable signals under such conditions is crucial for a broad applicability in protein identification.

By evaluating related proteins, we observed that translocation kinetics were strongly influenced by interactions between sequence-specific properties of the protein and the pore. Aerolysin K238A demonstrated promising potential for protein identification when coupled with machine learning algorithms. By optimizing the applied voltage, we achieved 80% classification accuracy for seven proteins from the same family, which shared 38–70% pairwise sequence identities. This highlights the potential of the nanopore system for high-accuracy, label-free protein identification. Looking forward, the primary bottleneck for scaling nanopore sensing to large analyte libraries lies in the physical resolution of the sensor rather than computational limitations, as classification algorithms are already well-established. By demonstrating sufficiently distinct physical “fingerprints” to resolve closely related analytes, this work provides a crucial step toward generating the high-quality data necessary to leverage more advanced machine learning architectures. While a fingerprinting approach cannot directly provide the analyte’s sequence, its ability to operate without enzymatic processing or chemical labeling, combined with machine learning-based classification, positions it as a practical and efficient tool.

Our findings suggest that the sequence-specific current patterns observed in aerolysin may reflect a combination of volume and positive charge distribution along the protein sequence. This model advances rational explanation for the observed signal patterns and offers a foundation for future advancements. With continued development, like identifying additional factors that influence the current output and translocation kinetics, or engineer/design pore architecture to accommodate less amino acids and therefore higher resolution, it may become feasible to predict nanopore fingerprints from protein sequences or, conversely, infer *de novo* sequence information directly from single-molecule signals, shedding light on previously uncharted biology.

## Supplementary Material



## Data Availability

All data are available in the main text or the Supporting Information. The databases for protein identification are available on Zenodo (10.5281/zenodo.17534056).

## Notes

Materials, methods, additional discussions, supporting tables, and figures (PDF)
