# Supplementary material for: Single-Molecule Fingerprinting of Unlabeled Full-Length Proteins Using an Aerolysin Nanopore
Source: J Am Chem Soc. 2026 Jun 29;148(27):28179–89. doi: 10.1021/jacs.6c01018 (PMC13387514; doi:10.1021/jacs.6c01018)
Supplement: Supplementary file 1 [file ja6c01018_si_001.pdf]

Supplementary information for

Single-molecule fingerprinting of unlabeled full-length proteins using an aerolysin nanopore

Verena Rukes[1,2], Evita Norkute[1], Georges Barnikol[3], Jingze Duan[1,4], Jiajie Gao[1], Chan Cao[1*]

[1]*Department of Inorganic and Analytical Chemistry, Chemistry and Biochemistry, University of Geneva; 1211 Geneva, Switzerland*

[2]*Institute of Bioengineering, School of Life Science, École Polytechnique Fédéralede Lausanne (EPFL); 1015 Lausanne, Switzerland*

[3]*Institute of Chemical Sciences and Engineering, School of Basic Sciences, EPFL; 1015 Lausanne, Switzerland*

[4]*Academy for Advanced Interdisciplinary Studies, Peking University; 100871 Beijing, China*

This file includes:

- Methods
- Supplementary Notes 1-4
- Supplementary Tables 1-8
- Supplementary Figures 1-24

Methods

Materials

Aerolysin K238A was produced and activated as described previously[1].

Turandot protein sequences were cloned into pET29b vectors, which were ordered from GenScript. Production was performed as described previously[2] by the Protein Production and Structure Core Facility at EPFL. TotA, TotB, TotC, TotX, TotZ, TotCpos, and TotCneg were expressed with a C-terminal His tag, while TotM and TotF were produced with C-terminal MBP-His constructs. A TEV cleavage site was included to allow removal of either tagging strategy. The plasmid encoding the protein of interest was transformed into Escherichia coli BL21 (DE3) cells. A 1L culture of Terrific Broth (TB) supplemented with kanamycin was inoculated and grown at 37°C until the optical density at 600 nm (OD600) exceeded 0.6. Protein expression was induced with 0.5 mM isopropyl β-D-1-thiogalactopyranoside (IPTG), and the culture was incubated at 18°C overnight. Two main buffers were used for the purification process: Buffer A – 700 mM NaCl, 20 mM 2-(4-(2-hydroxyethyl)-1-piperazinyl)ethanesulfonic acid (HEPES), pH 7.5.; Buffer B – 700 mM NaCl, 20 mM HEPES, pH 7.5, 500 mM Imidazole. For dialysis, a buffer containing 20 mM HEPES, pH 7.5, and 300 mM NaCl was used. The bacterial pellet was resuspended and lysed. The supernatant was incubated with 5 mL of HisPur Ni-NTA Resin beads (Thermo Scientific) at 4°C for 2 hours and eluted on a gradient from buffer A to 100% buffer B. Fractions containing the protein of interest were pooled and subjected to overnight cleavage with 0.7 mg SuperTEV protease at 4°C during dialysis. Cleaved proteins underwent reverse Ni-NTA chromatography to remove the protease and cleaved tag. The purified and concentrated protein samples were flash-frozen and stored at -80°C for future use.

Nanopore sensing experiments

Nanopore measurements were carried out in the indicated buffer conditions containing 2 M GdmCl (Sigma) or 3 M GdmCl buffered either with 10 mM tris(hydroxymethyl)aminomethane to pH 7.5 or with 10 mM citrate to pH 4.0. Lipid bilayers of 1,2-diphytanoyl-sn-glycero-3-phosphocholine (DPhPC, Avanti polar Lipids) were formed on the 50 µm apertures of MECA 4 Recording Chips (Ionera, Germany) and small amounts of activated aerolysin K238A pore were added to the *cis* chamber to allow single pore insertion. Data was acquired at 100 kHz using an Orbit mini device (Nanion technologies, Germany). Temperature control of the instrument was set to 25°C for all measurements. Each experiment was carried out several times and with > 5 individual pores. During condition optimization, TotA was measured at 0.5 µM initially. Subsequent measurements were carried out at 1 µM analyte concentration.

Exceptionally, measurements for the mixture experiment were carried out at 0.1 µM of each analyte for the reference and mixture recordings.

Reversal potential measurements

To obtain the reversal potential, measurements were carried out on Axonpatch 200B (Molecular Devices) using polystyrene cups (Warner Instruments). 1 µl of 25 mg/ml DPhPC in hexane (Sigma) was used to pretreat the cup. Following evaporation, 1225 µl of 0.5 M electrolyte were added to both the *cis* and *trans* chamber. Electrodes were installed with salt bridges and membranes were formed using 8 mg/ml DPhPC in decane (Sigma). Small amounts of activated aerolysin K238A were added to the *cis* chamber. Following a pore insertion, the pipette offset was used to ensure that 0 pA were measured at 0 mV in symmetric buffer conditions (0.5 M KCl). Subsequently, 525 µl of the *cis* buffer were exchanged for buffer at the same pH with 4 M electrolyte for a final 2 M in *cis* versus 0.5 M in *trans*. Following carful mixing and several minutes of equilibration time, the voltage was altered in 2 mV steps within the range of ± 40 mV. As the current depended linearly on the voltage in this regime, a linear regression allowed to precisely extract the voltage at 0 pA, which is known as reversal potential (V_R). Activity coefficients[3,4] were used to convert concentrations to activity a. The reversal potential V_R was used to calculate P^-/P^+ with the Goldman–Hodgkin–Katz flux equation (Equ. 1) [5].

$$\frac{P^-}{P^+} = \frac{[a_{cation}]_{cis} \cdot \exp\left(-\frac{eV_R}{k_B T}\right) - [a_{cation}]_{trans}}{[a_{anion}]_{cis} - [a_{anion}]_{trans} \cdot \exp\left(-\frac{eV_R}{k_B T}\right)} \qquad \text{Equ. 1}$$

With the Boltzmann constant k_B, elementary charge e and the absolute temperature T. Final values are averaged over results from at least 4 individual pores (Fig. S1).

Pretreatment of protein analytes

Samples were equilibrated in 6 M GdmCl and heated to 95°C for 10 min before being measured in the nanopore system or in Circular Dichroism.

Circular dichroism

Circular dichroism (CD) measurements were performed using a Chirascan™ V100 (Applied Photophysics) spectropolarimeter. Samples were analyzed in quartz cuvettes with a path length of 0.1 cm. Spectra were recorded from 210 to 280 nm at 25°C. Each spectrum was an

average of 3 accumulations. The air baseline was automatically subtracted from all measurements by the instrument. Buffer spectra were recorded separately and used as backgrounds to be subtracted for each corresponding sample. TotA was measured following the pre-equilibration protocol at 10 µM final concentration in all conditions. Data processing was performed in python. The results are presented as mean residue ellipticity (deg·cm^2·dmol^{-1}) with consideration of protein concentration, path length, and number of amino acid residues. Signal analysis was performed with BeStSel[6].

<u>Data analysis</u>

The event-current representing single molecule interactions was extracted from raw traces in python. Briefly, the traces were filtered to 15 kHz with an eighth order low pass Bessel filter and segmented at each deviation in voltage. For each segment, the open pore current (OPC, I_0) and its standard deviation (sigma) were determined. Regions in the trace that correspond to events were defined as starting when the current dropped below 10 sigma from the OPC and ending when the current had recovered to 1 sigma below OPC. Events were extracted from the unfiltered raw traces using the regions determined in filtered traces. The gradient of the datapoints was thresholded at the beginning and end of the event to exclude datapoints from the falling and rising regions of the measured current. The current of the open pore state from each segment is used to normalize the current of the events resulting in the average relative current in % of the open state. Similarly, the standard deviation of the events is normalized by the standard deviation of the open pore state, to avoid biases from the membrane noise.

To exclude background and outlier data, events with less than 15% mean relative residual current and dwell time of 0.25 – 100000 ms were selected and further cleaned using the Isolation Forest algorithm from the Scikit-learn library. For this, the mean, mode, and median of the relative current, as well as its standard deviation and the logarithmic dwell time of the event were used as event features. Dwell times of the populations were fitted with an exponentially modified gaussian distribution or the Fokker-Planck equation[7] (Equ. 2) with *L* the contour length of each protein plus the length of the pore (estimated at 10 nm), the dwell times *t*, the diffusion coefficient *D*, and the drift velocity *v*.

$$F(t) = \frac{L}{\sqrt{3\pi D t^3}} \cdot \exp\left(-\frac{(L - vt)^2}{4Dt}\right) \qquad \text{Equ. 2}$$

Since the equation overly simplifies the system, fitted *D* and *v* values were not considered reliable and are thus not discussed. The equation was used merely to extract each distributions' maximum and half widths.

For statistical analysis, the SciPy package in python was used. Linear regressions and pearson correlations were performed using the default settings of the linregress and pearsonr function, respectively.

Time normalized overlay plots were produced as done in our previous study[8]. Briefly, currents (I/I_0 in %) of randomly selected events were normalized to a length of 200 data points by dividing them into 200 equal chunks and averaging them. In cases of events with less than 200 data points, they were interpolated using numpy's interp function.

Machine learning

For identification, additional features were extracted from the event data (listed in SI Table S2). The current of each event was Bessel filtered to 10 kHz and divided into 8 equally long parts, or three equally long parts as indicated in SI Table S2. Features were also extracted from the power spectrum of each event by segmenting the spectrum into 8 parts and averaging it similar as in a previous study[9]. Additionally current values were separated into two clusters using a gaussian mixture model and averaged, yielding a low current and high current average for each event.

The maximum equal number of events were used to fit a random forest classifier from the scikit-learn library and only features with a mean decrease in impurity of over 0.01 were selected for further classification (SI Fig. S11). Classification algorithms from the scikit-learn library were cross validated and the top three performing classifiers (Random Forest, Bagging, and Extra Trees Classifier) were used in a voting classifier. To estimate the quality of protein identification, accuracy was estimated in 10 rounds and averaged. For each round, the equal number of events was randomly sampled from each protein analyte, followed by a split that left 20% of the data points for testing. A pipeline of standard scaler and voting classifier was built and trained on the training data set.

For Fig. 4C all combinations of Tot-family proteins were explored. Here, accuracies for each combination are averaged over three rounds sampling, training and testing were performed for each approach of classification. Approaches i) and ii) were the same as previously described, except that for ii) only dwell time and current were used. Approach iii) uses the DummyClassifier form the scikit-learn library with strategy "stratified".

All-atom MD simulation

Building MD simulation system

We prepared the molecular dynamics (MD) simulation systems using the cryo-EM structure of wild-type aerolysin (PDB: 9FM6)[10]. CHARMM-GUI was used to generate the mutant K238A, configuration, and topology of the simulation systems[11]. The parameter files were created using the CHARMM36m force field[12]. Partial charges for Gdm^+ were slightly adjusted to 0.679 for its carbon, 0.438 for its hydrogen, and -0.769 for its nitrogen atoms. The PPM server was used to reorient the aerolysin structure, ensuring that its transmembrane region was correctly positioned in a lipid bilayer[14]. The protein was embedded in a PhPC bilayer. Next, we solvated the protein-membrane complex in a water box using the TIP3 water model. Cations and anions were added to achieve a concentration of 3 M while counterions were introduced to neutralize the system. Further details of simulation systems are shown in SI Table S1.

All of the MD simulations were performed with GROMACS 2023.1[15]. The REDUCE program in AMBER was used to add hydrogens to the original PDB files and determine the protonation state at pH 7.5[16]. To accurately calculate the protonation state at low pH, we used the APBS server for pH 4.0 systems to correct the protonation state[17].

We used the steepest descent algorithm to achieve energy minimization. Then, following up with a two-stage equilibration, a 0.4 ns NVT equilibration simulation with harmonic restraint was applied to the protein molecule (force constants of 4000 $kJ \cdot mol^{-1} \cdot nm^{-2}$ on the backbone and 2000 $kJ \cdot mol^{-1} \cdot nm^{-2}$ on the side chains), and a 20 ns NPT equilibration simulation with gradually decreased restraint (from 2000 to 100 $kJ \cdot mol^{-1} \cdot nm^{-2}$ on the backbone and from 1000 to 50 $kJ \cdot mol^{-1} \cdot nm^{-2}$ on the side chains). During the equilibration processes, planar restraints were used to keep the positions of lipid head groups along the membrane-normal direction.

The simulation temperature of the system was set to 300 K. The time step was 2 fs. The cubic periodic boundary condition was used during the simulations, and the van der Waals interaction was switched off from 10 to 12 Å. The long-range electrostatic interactions were calculated using the particle mesh Ewald (PME) method[18].

To investigate the ion transport properties of the pore and understand its corresponding ion selectivity feature, we applied a 100 mV transmembrane potential to the channel and analyzed the ion translocation process across the pore.

Analysis of electrostatic potential map

We calculated the surface electrostatic potential of the protein with APBS electrostatic plugin in VMD[17].

Analysis of the binding distribution of ions

We used the HOLE program to get the inner radius of the aerolysin K238A structure[19].To analyze the binding of ions to the pore lumen we only consider the ions that enter the protein channel within the trajectory. For each type of ion and position along the pore lumen we then sum the time during which binding occurred. Binding was identified, when the distance between the central carbon atom of a Gdm^+ ion – or for other ions the exact position of the atom – and the pore wall was less than 4 Å. This is similar to what has been defined previously[20]. For the ions that entered the pore, the ratio of each bound ion was calculated by dividing the accumulated binding time along the Z coordinate by the total time of the trajectory. Final distributions along the Z coordinate were averaged over the ions that entered the pore.

Calculation of simulated EOF

To quantify the EOF in our simulated system, we separately counted the number of water molecules passing through the pore from the *trans* to *cis* direction and from the *cis* to *trans* direction. To obtain the rate of water molecules transported over time, a least-squares linear fit to the time-dependent profile of the net cumulative number of water molecules passing through the pore was performed.

Current interpretation

To interpret current signatures, each amino acid in a sequence was first replaced by its charge or volume [21]. Charges were calculated based on the pH and pKa[22] of the residues and terminal groups. Terminal charges were added to the first and last amino acid and all negative values were set to 0 to only consider the positive charges. The sum of either positive charges or volume of each set of 30 subsequent amino acids along the sequence was calculated. The summed volume of the analyte portion (V_{30aa}) was further converted into relative residual volume as $\frac{(V_{pore}-V_{30aa})\cdot 100\%}{V_{pore}}$ using the approximate pore volume $V_{pore}=\pi r^2 h$. The pore volume was calculated using the average water accessible radius of the pore ($r = 1.15\ \mathrm{nm}$) and the height of the aerolysin barrel ($h = 10\ \mathrm{nm}$). To calculate the grand average hydropathy (GRAVY) the Kyte-Doolittle scale for hydropathy was used [23].

To generate consensuses of signals from each analyte at each voltage, all events after data cleaning were filtered with a Sawitzky-Gloay filter. A 10th order polynomial fit was applied to each entire event and the output was down sampled to 100 points. Subsequently, the median across all events at each of the 100 points was calculated yielding the consensus. The standard deviation at each point was extracted to later calculate weighted DTW distances[24].

To estimate the distance between the parameters stemming from sequences (charges and residual volumes) and the consensuses, we performed scaling with the minimal (min) and maximal (max) value across all analytes at a given voltage $\frac{trace-min}{max-min}$. Subsequently, we calculated the DTW distance weighing[24] the consensus by the inverse of the standard deviation.

A prediction was generated as $Prediction = w_{(+)q} \cdot (+)q + w_{V_{res}} \cdot V_{res}$. With $(+)q$ the trace of positive charges in sliding windows along the analytes sequence and V_{res} the trace of corresponding residual volumes. Weights $w_{(+)q}$ and $w_{V_{res}}$ were optimized using the minimize function from SciPy. As a loss function, the sum of weighted DTW distance between prediction and the consensus over all analytes was used. To avoid bias, each distance was normalized by the length of the prediction (which depended on the analyte length). Weights were optimized for data from each recording voltage separately. The predictions are min-max normalized across the analytes both while optimizing the weights and before calculating distances to the consensuses.

SI Note 1: event populations

At higher voltages a second, well separated population of events is obtained when measuring TotA in 2 M or 3 M GdmCl at pH 7.5, as seen in scatterplots SI Fig. S4. This additional population has lower residual current and larger dwell times than the main population observed at lower voltage. Different populations from the same analyte can be associated with different entry conformations or orientations to the pore.

At pH 4.0, one population is obtained for all voltages. The population is elongated towards increasing dwell time and deeper blockage at high voltages. However, as seen in dwell time histograms (SI Fig S5), these elongated events typically make up a small portion of the population. It is possible that the higher uniformity at pH 4.0 stems from the more unidirectional capture due to the changed protonation states of the terminal groups. As shown in Fig. 5D the C-terminus is more negatively charged than the N-terminus at pH 4.0 for all investigated members of the Tot protein family.

SI Note 2: Translocation velocities

Translocation velocities (Fig. 3E, proteins length divided by the fitted dwell time) depended roughly linearly on the electric field $E = \frac{\Delta V}{d}$, with the voltage drop ΔV over the length of the pore ($d \approx$ 10 nm). The transport in this system is driven by the EOF against the EPF and we therefore observe $v_{total} = v_{EOF} + v_{EP}$. The electroosmotic flow velocity v_{EOF} in a given biological nanopore system is directly proportional to the current I [25], which in turn depends linearly on the voltage in the measured regime (SI Fig. S4). The electrophoretic velocity has been described as $v_{EP} = \mu_{EP} \cdot E = \mu_{EP} \cdot \frac{\Delta V}{d}$ with the electrophoretic mobility μ_{EP}. Thus, the observed linear dependence of the velocity on the electric field is in alignment with expectations.

SI Note 3: Influence of positive charges on signal patterns

We discovered an effect of the analytes' positive charges on the residual current of translocation events through designed protein analytes (SI Fig. S21). The unstructured loop of TotC was replaced with two control sequences: one with more negative charges (TotCneg), and another with more positive charges (TotCpos). Their charge distributions at pH 4.0 are shown in SI Fig. S21A. We found a higher current level in the signals for TotCpos (SI Fig. S21B) which led to larger currents of the population average across all voltages for TotCpos translocations compared to TotC and TotCneg (SI Fig. S21C). TotCneg behaved very similarly to TotC, suggesting that the positive charges produced a much more pronounced effect than

negative ones in our system. This could be explained by the asymmetry of the ion transport in the GdmCl pH 4.0 system, where anions are more mobile than cations. Introducing additional positive charges inside the pore can thereby increase anion (Cl^-) fluxes and the total current. A similar effect was observed in the open pore current, which increased when the pore lumen was more positively charged at pH 4.0 with larger ion selectivity compared to pH 7.5 (SI Fig. S6). As negatively charged aa occupy the pore, they could only neutralize part of the asymmetry in ion transport and should therefore not lead to a current increase. Additionally, the negative charges of aa at pH 4.0 are merely partial, likely contributing to their limited impact on the current.

SI Note 4: Signal interpretation for N-terminal capture

As shown in Fig. 5D the C-terminus is more negatively charged than the N-terminus for all Tot family proteins. This gives a good indication that they should be primarily captured by the C-terminus in our system. Nonetheless, we have performed additional signal pattern analysis for N-terminal capture SI Fig. S22. Contrary to C-terminal capture, the charges agree better than the volumes for N-terminal capture (SI Fig. S22 A and C). Charges and volumes can be combined to a prediction, which however is less consistent in improving the agreement with the consensus than the predictions for C-terminal capture (Fig. 5E).

SI Tables

Table S1. MD simulation systems of aerolysin K238A in different buffer and pH.

Trajectory label	pH	Ions	Box size (Å)	Atom number	Trajectory time (ns)
Traj-1	4.0	3 M GdmCl	199×199×150	610,531	450
Traj-2	7.5	3 M GdmCl	198×198×150	611,415	450
Traj-3	4.0	3 M KCl	195×195×151	583,387	300
Traj-4	7.5	3 M KCl	197×197×149	573,545	200

Table S2. Number of events in condition optimization experiments

**Analyte**	TotA			
**Condition**	3 M GdmCl pH 7.5	3 M GdmCl pH 4.0	2 M GdmCl pH 7.5	2 M GdmCl pH 4.0
**100 mV**	5668	19857	4721	6822
**120 mV**	4327	19697	8809	8500
**150 mV**	9525	58977	5438	7462
**170 mV**	11086	73361	6550	6739
**190 mV**	5690	53829	1897	9840
**210 mV**	4120	24597	1730	2461

Table S3. Features of the protein analytes. Charges calculated using protein tool[26]. GRAVY is based on the Kyte-Doolittle hydropathy scale[23].

	TotA	TotB	TotC	TotF	TotM	TotX	TotZ	TotCpos	TotCneg
Length (aa)	115	123	114	111	114	126	130	119	119
Charge at pH 4.0	8	15.6	10.1	11.8	9.92	14.9	-1.062	15.3	-0.4
GRAVY	-0.41	-0.57	-0.55	-0.25	-0.37	-0.79	-0.47	-0.96	-0.91
Molecular weight (Da)	12828	14132	12901	12531	13204	14944	14517	13345	13500

Table S4: Number of events measured in 3 M GdmCl pH 4.0

**Analyte**	TotB	TotC	TotF	TotM	TotX	TotZ	TotCpos	TotCneg
**Condition**	3 M GdmCl pH 4.0							
**100 mV**	2906	3510	6932	3710	2647	1441	2849	5995
**120 mV**	3170	2654	5018	2785	2423	1557	2472	3126
**150 mV**	3935	3254	1041	860	986	599	5934	4111
**170 mV**	1898	1499	722	841	1072	680	5483	3025
**190 mV**	1868	2701	1386	626	1193	710	5841	2418
**210 mV**	3574	1677	1281	695	1087	516	4373	2835

Table S5. Results from linear regression of translocation velocities corresponding to figure 3E.

	TotA	TotB	TotC	TotF	TotM	TotX	TotZ
**Slope (nm^2 mV^{-1} ms^{-1})**	0.971	0.334	1.004	0.312	0.643	0.444	0.505
**Intercept y axis (nm ms^{-1})**	-4.895	2.77	-2.76	-1.115	-5.193	1.277	3.126

Table S6. P-values of Pearson correlations shown in figure 3F and SI figure S15.

**Voltage**	**Hydropathy-Dwell time**	**Charge-Dwell time**	**Length-Dwell time**
100	0.22	0.57	0.23
120	0.13	0.78	0.2
150	0.16	0.81	0.28
170	0.18	0.58	0.37
190	0.29	0.41	0.59
210	0.32	0.44	0.5

Table S7: Number of events measured in 3 M GdmCl pH 4.0 used as labeled datasets for machine learning.

**Analyte**	TotA	TotB	TotC	TotF	TotM	TotX	TotZ	**Used in ML**
**Condition**	3 M GdmCl pH 4.0							
**100 mV**	3401	1744	2106	4159	2226	1588	865	865
**120 mV**	2596	1902	1592	3011	1671	1454	934	934
**150 mV**	5715	2361	1952	625	516	592	359	359
**170 mV**	6652	1139	899	433	505	643	408	408
**190 mV**	3414	1121	1621	832	376	716	426	376
**210 mV**	2472	2144	1006	769	417	652	310	310

Table S8. event features used for machine learning classification. Except for feature 0 (dwell time) all features are described as calculations on the extracted current that was normalized by the open pore current and filtered to 10 kHz. For features 7 and 8 current data point were divided into two clusters using a gaussian mixture model. “Diff mean xy” describes the difference between the mean of segments x and y from events segmented into 8 equally long segments.

0	logarithmized dwell time	31	standard deviation part 7/8	62	max part 2/8
1	mean	32	standard deviation part 8/8	63	max part 3/8
2	mode	33	mean power spectrum part 1/8	64	max part 4/8
3	median	34	mean power spectrum part 2/8	65	max part 5/8
4	standard deviation	35	mean power spectrum part 3/8	66	max part 6/8
5	skewness	36	mean power spectrum part 4/8	67	max part 7/8
6	kurtosis	37	mean power spectrum part 5/8	68	max part 8/8
7	mean of lower current cluster	38	mean power spectrum part 6/8	69	skewness part 1/8
8	mean of higher current cluster	39	mean power spectrum part 7/8	70	skewness part 2/8
9	median part 1/8	40	mean power spectrum part 8/8	71	skewness part 3/8
10	median part 2/8	41	mean absolute gradient	72	skewness part 4/8
11	median part 3/8	42	mean gradient	73	skewness part 5/8
12	median part 4/8	43	mean abs gradient 1/3	74	skewness part 6/8
13	median part 5/8	44	mean abs gradient 2/3	75	skewness part 7/8
14	median part 6/8	45	mean abs gradient 3/3	76	skewness part 8/8
15	median part 7/8	46	diff mean 12	77	mode part 1/8
16	median part 8/8	47	diff mean 23	78	mode part 2/8
17	mean part 1/8	48	diff mean 34	79	mode part 3/8
18	mean part 2/8	49	diff mean 45	80	mode part 4/8
19	mean part 3/8	50	diff mean 56	81	mode part 5/8
20	mean part 4/8	51	diff mean 67	82	mode part 6/8
21	mean part 5/8	52	diff mean 78	83	mode part 7/8
22	mean part 6/8	53	min part 1/8	84	mode part 8/8
23	mean part 7/8	54	min part 2/8	85	correlation part 1/3
24	mean part 8/8	55	min part 3/8	86	correlation part 2/3
25	standard deviation part 1/8	56	min part 4/8	87	correlation part 3/3
26	standard deviation part 2/8	57	min part 5/8		
27	standard deviation part 3/8	58	min part 6/8		
28	standard deviation part 4/8	59	min part 7/8		
29	standard deviation part 5/8	60	min part 8/8		
30	standard deviation part 6/8	61	max part 1/8		

SI Figures

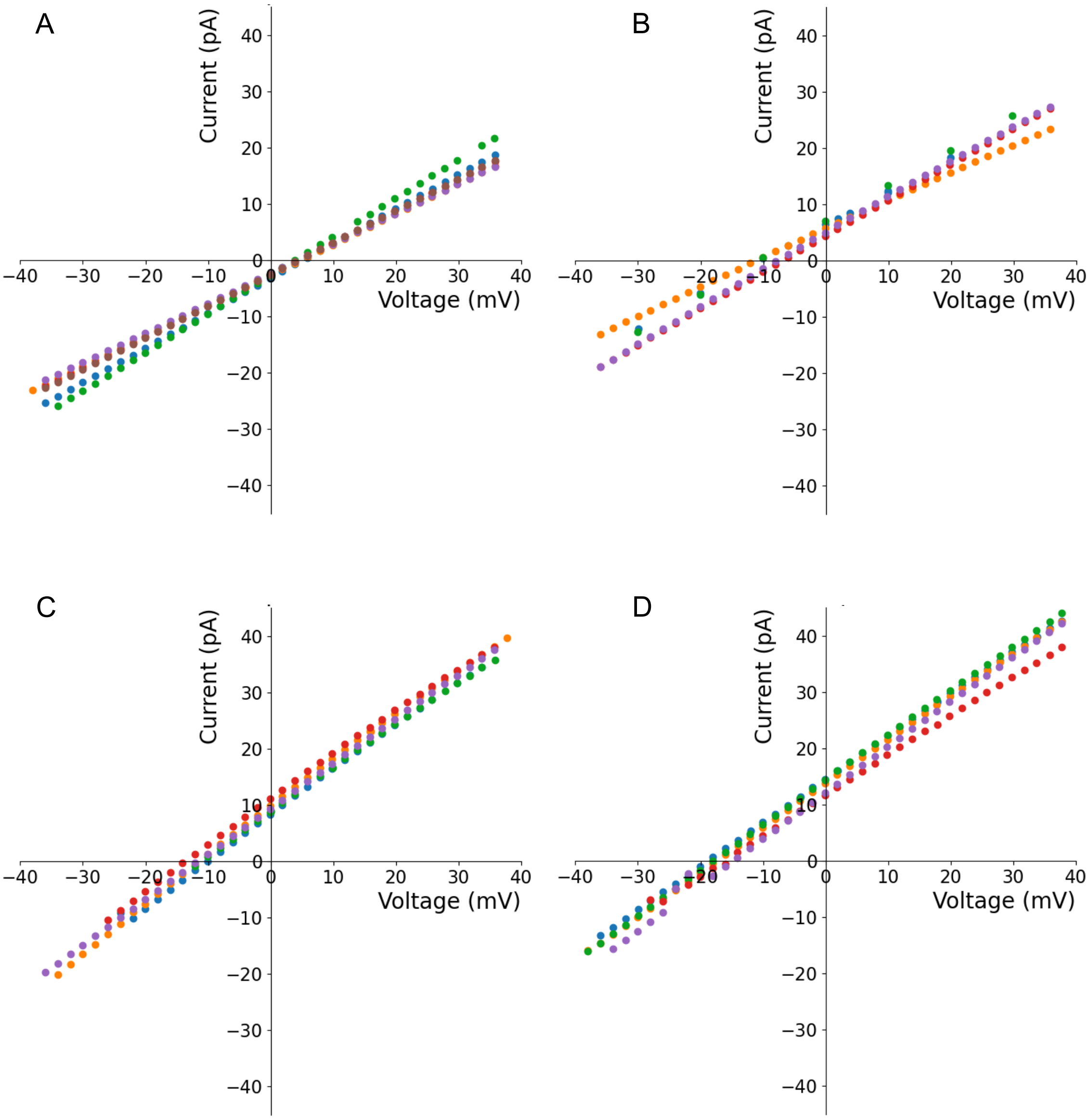

Figure S1. Current versus voltage data at 2 M *(cis)* vs 0.5 M *(trans)* electrolyte measured with aerolysin K238A *in* KCl at pH 7.5 (A), GdmCl at pH 7.5 (B), KCl at pH 4.0 (C), and GdmCl at pH 4.0.

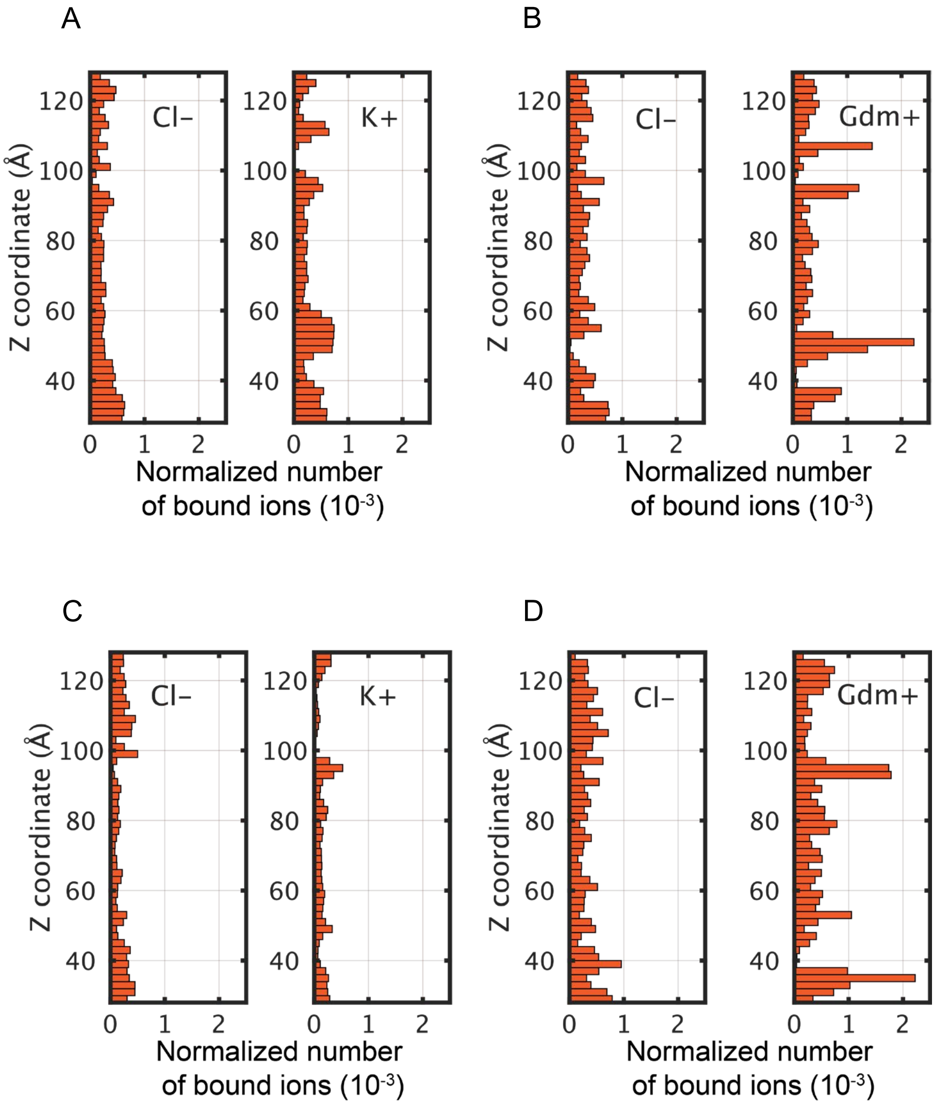

Figure S2. MD results displaying the duration of ions binding to the surface of aerolysin K238A normalized by the number of interacting ions. (A) 3M KCl at pH 7.5, (B) 3M GdmCl at pH 7.5, (C) 3M KCl at pH 4.0, (D) 3M GdmCl at pH 4.0.

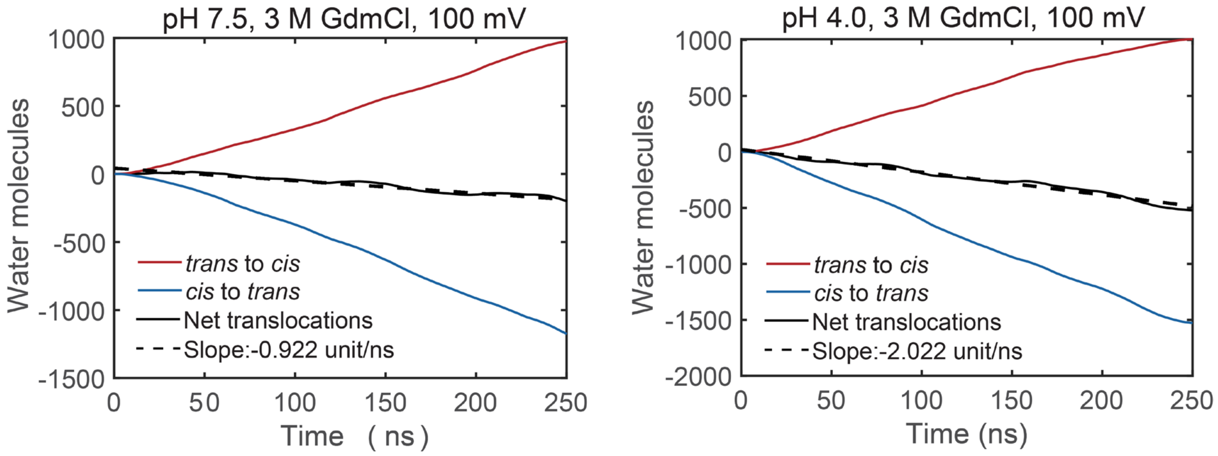

Figure S3. MD results for calculations of the EOF. Cumulative number of water molecules that translocated the aerolysin K238A nanopore from *trans* to *cis* (red) and *cis* to *trans* (blue) with the net number shown in black. Systems at pH 7.5 (A) and pH 4.0 (B) in 3 M GdmCl at 100 mV applied to the *trans* side. A linear regression was performed on the net water transport (dashed lines) yielding slopes of –0.922 and –2.022 units/ns or water molecules/ns at pH 7.5 and pH 4.0, respectively.

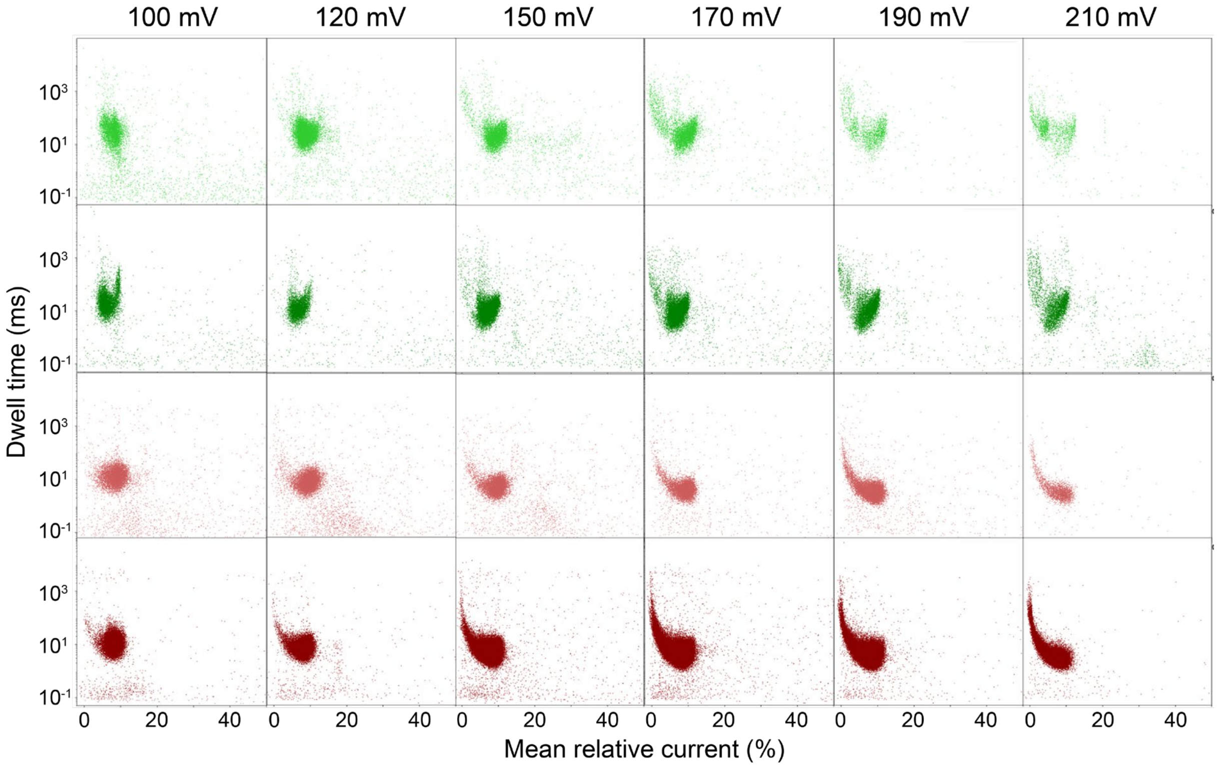

Figure S4. Scatter plots of individual single events from TotA represented by event duration against of mean relative current. From top to bottom: 2 M GdmCl pH 7.5, 3 M GdmCl pH 7.5, 2 M GdmCl pH 4.0, 3 M GdmCl pH 4.0 at (left to right) 100 mV, 120 mV, 150 mV, 170 mV, 190 mV, 210 mV.

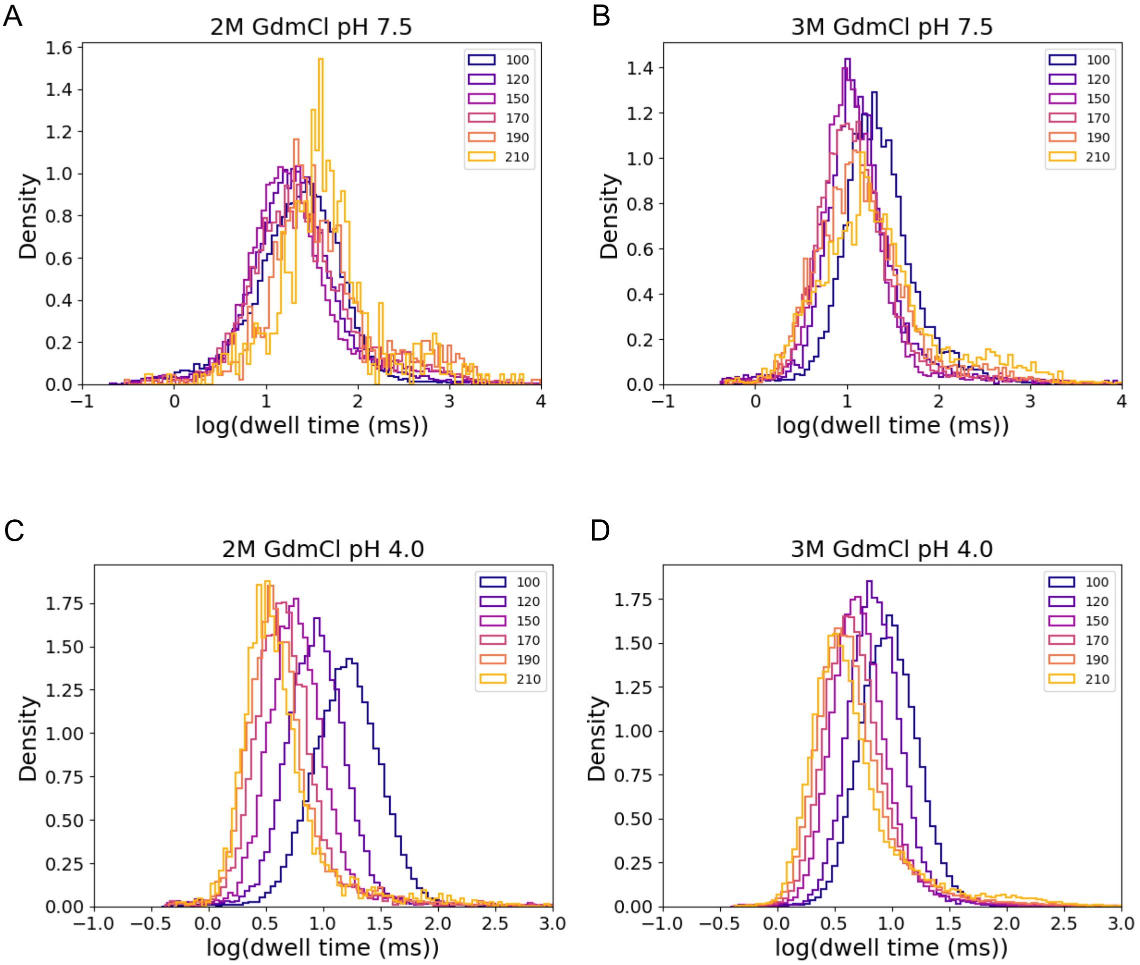

Figure S5. Histograms of logarithmized dwell times of TotA measured with aerolysin K238A in (A) 2 M GdmCl pH 7.5, (B) 3 M GdmCl pH 7.5, (C) 2 M GdmCl pH 4.0, and (D) 3 M GdmCl pH 4.0. Overlays are shown of the different voltages that were measured 100 mV (dark blue), 120 mV (purple), 150 mV (magenta), 170 mV (dark orange), 190 mV (orange), and 210 mV (yellow).

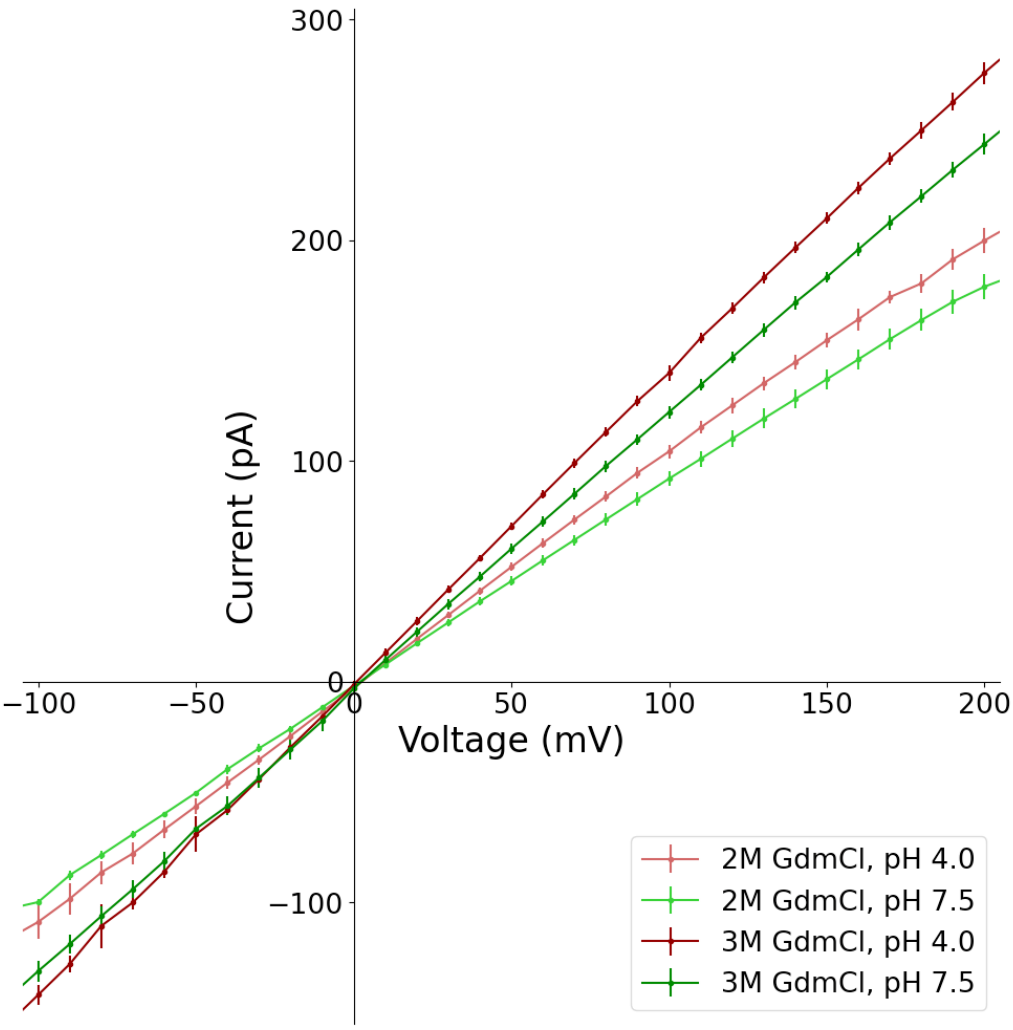

Figure S6. Open pore current of aerolysin K238A at various voltages in the indicated conditions.

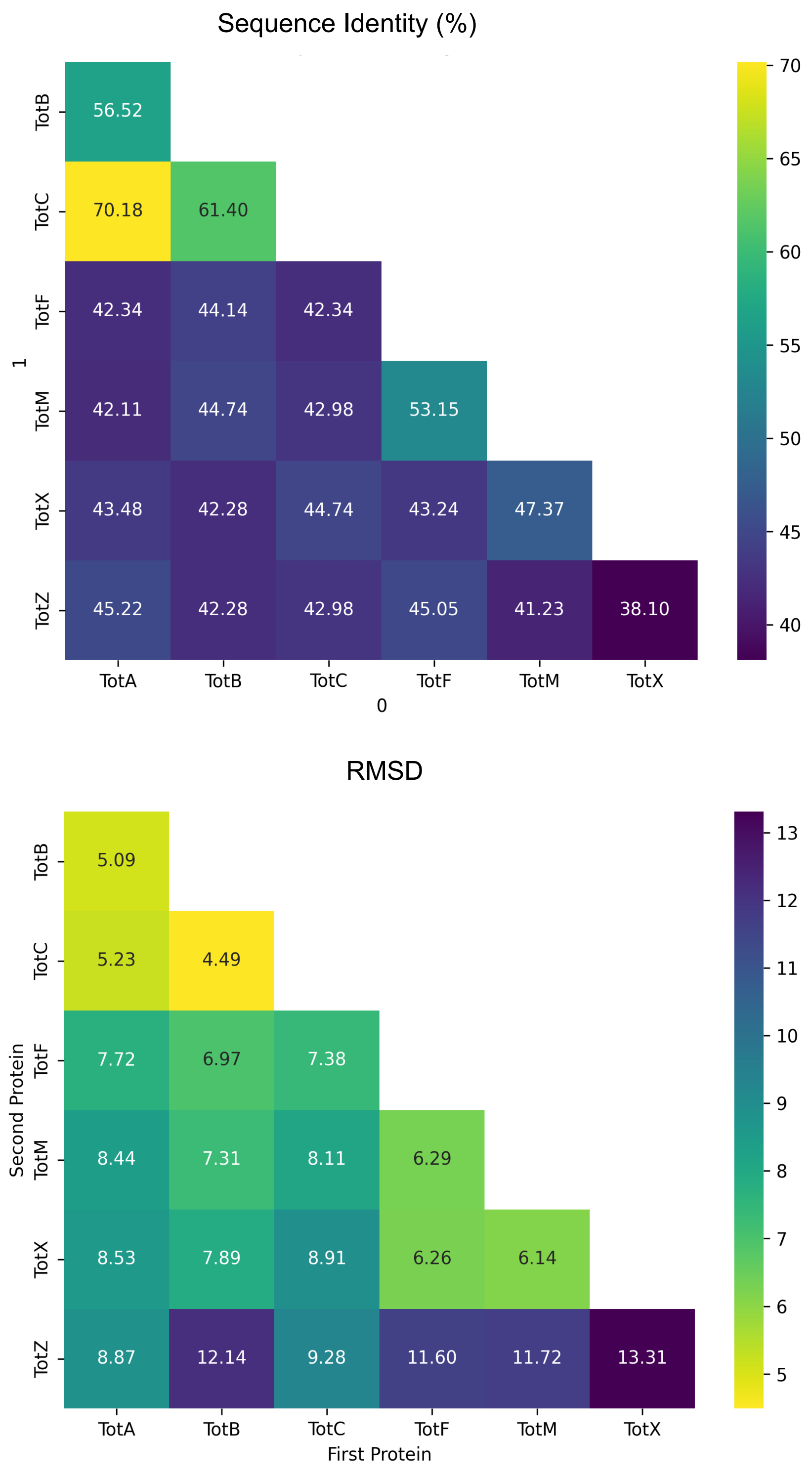

Figure S7. Sequence similarity (left) and structural similarities (right) calculated by Root mean square deviation (RMSD) of Tot family proteins

TotA:

MGYSDEDREADNLRIAEIIKNAQDDDSKINSTQELLDIYRRLYPSLTPEERESIDKFVNEHTDAIIID
GVPIQGGRKARIVGKIVSPGVKGLATGFFEELGSKLAQLFAGENLYF

TotB:

MYSNQERQRDSRRVAEIMRTSWDDNTKIKRIQELLTIYNRMAPSLRPDERARMDRFISGYTGEIM
VDGVPSQGGARRIFKKILSPAAKSVATGFFTELGASLASILTSWFPANTERNHENLYF

TotC:

MYSDEERESDSLRVAEIIRTSNDAESKINRTQELLDIFRRLTPTLSPEQREKIERSIQEHTDEILIDG
VPSQGGRKTKYVGKILSPVAQGLAVGFFEELGGSLSRLFTGENLYF

TotF:

MEHAQSDPEFTAKARQMLAVFGNSEVDRYTKSRNLPALIEFYEKYSSRLPLTVQDRTYANNVIRR
YRAHNNQQVDGVPAQGGVGVVFALLLPFAVSIVEGIAKAIRENLYF

TotM:

MENEDEFVTEKQRLFSVYGDSSVDEATKYRNIDSLVTFYDKYFTRLQLKPDLNTRAHDLLRRYKE
ENARVVLVDGTPAQGGFWLPLVKLLIVQLGVEIASEGVKRAIESENLYF

TotX:

MNTNSSSYEEHRNYLLNIFHNPFVNDSIKEKNIPQLIAFYQRYPTDVPLSDADRQQFERFIHDYRE
YRAVLVDGAPPQGGSFGNIFGHFLGRVGTRYISSLFNKKREERKSNHAYIIEDYNENLYF

TotZ:

MRMLDADRNRLQQLQIRSQQSADANTQVDIAYEVIGIYDKYKGQGGSNVLREAQLNSQVNDFK
RKTMVIDGVPAQGGVWGILGAIKKAADAVPDNVKKDAENLVKSSTKVLVRGIYDYLMGKMKHEN
LYF

TotCpos:

MYSDEERESDSLRVAEIIRTSNDAESKINRTQELLDIFRRLTPTLSPEQREKIERSIQEHTDEILIGG
NKSNRSRNKGQKRSKSKAKGQSPVAQGLAVGFFEELGGSLSRLFTGENLYF

TotCneg:

MYSDEERESDSLRVAEIIRTSNDAESKINRTQELLDIFRRLTPTLSPEQREKIERSIQEHTDEILIGD
DSDDGDDEDDSDDGDDEDDGGSPVAQGLAVGFFEELGGSLSRLFTGENLYF

Figure S8. Protein sequences of measured analytes.

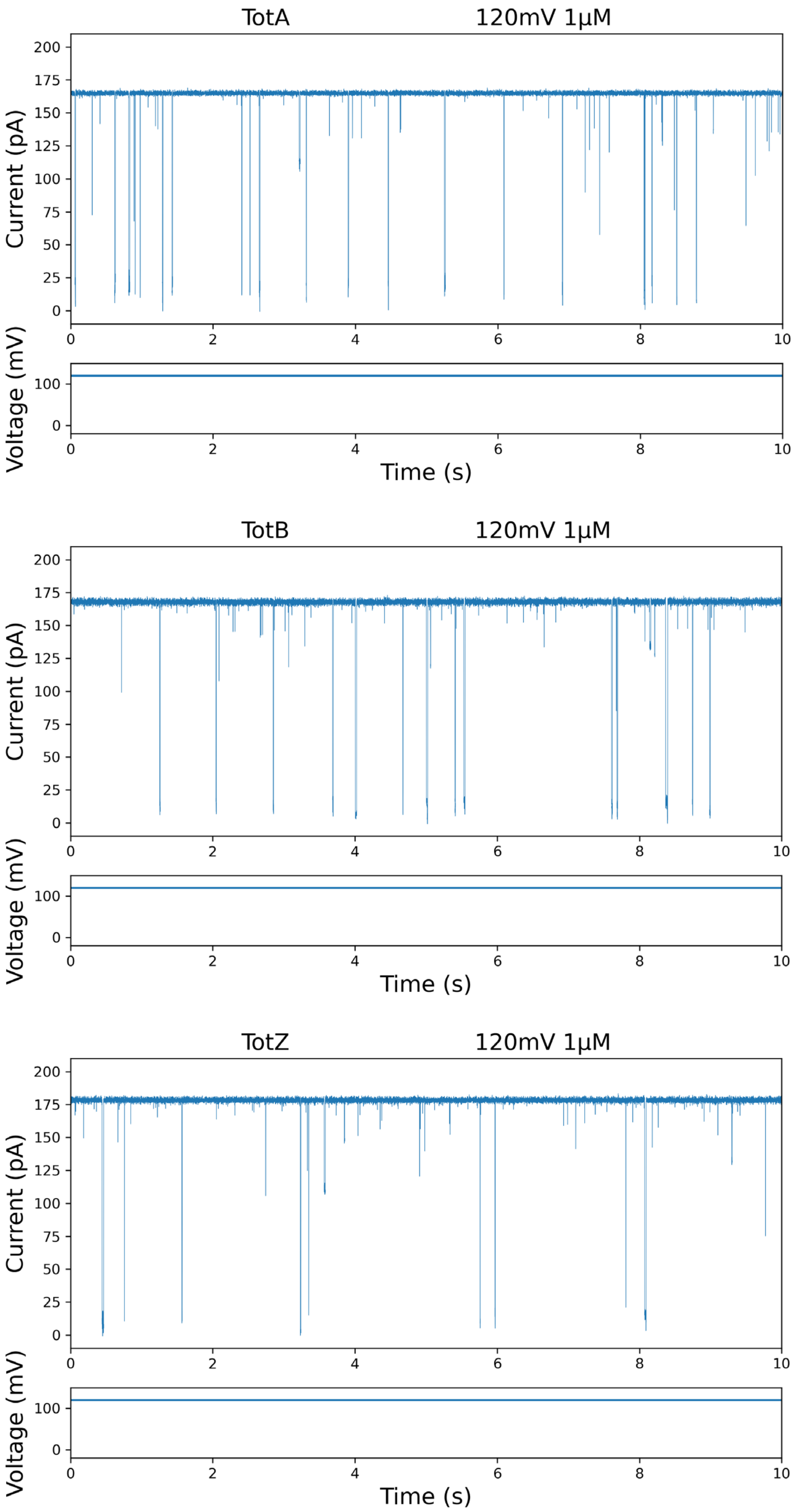

Figure S9. Example traces of TotA, TotB, and TotZ recorded in 3 M GdmCl at pH 4.0, 120 mV, filtered with 1 kHz for illustration purpose only.

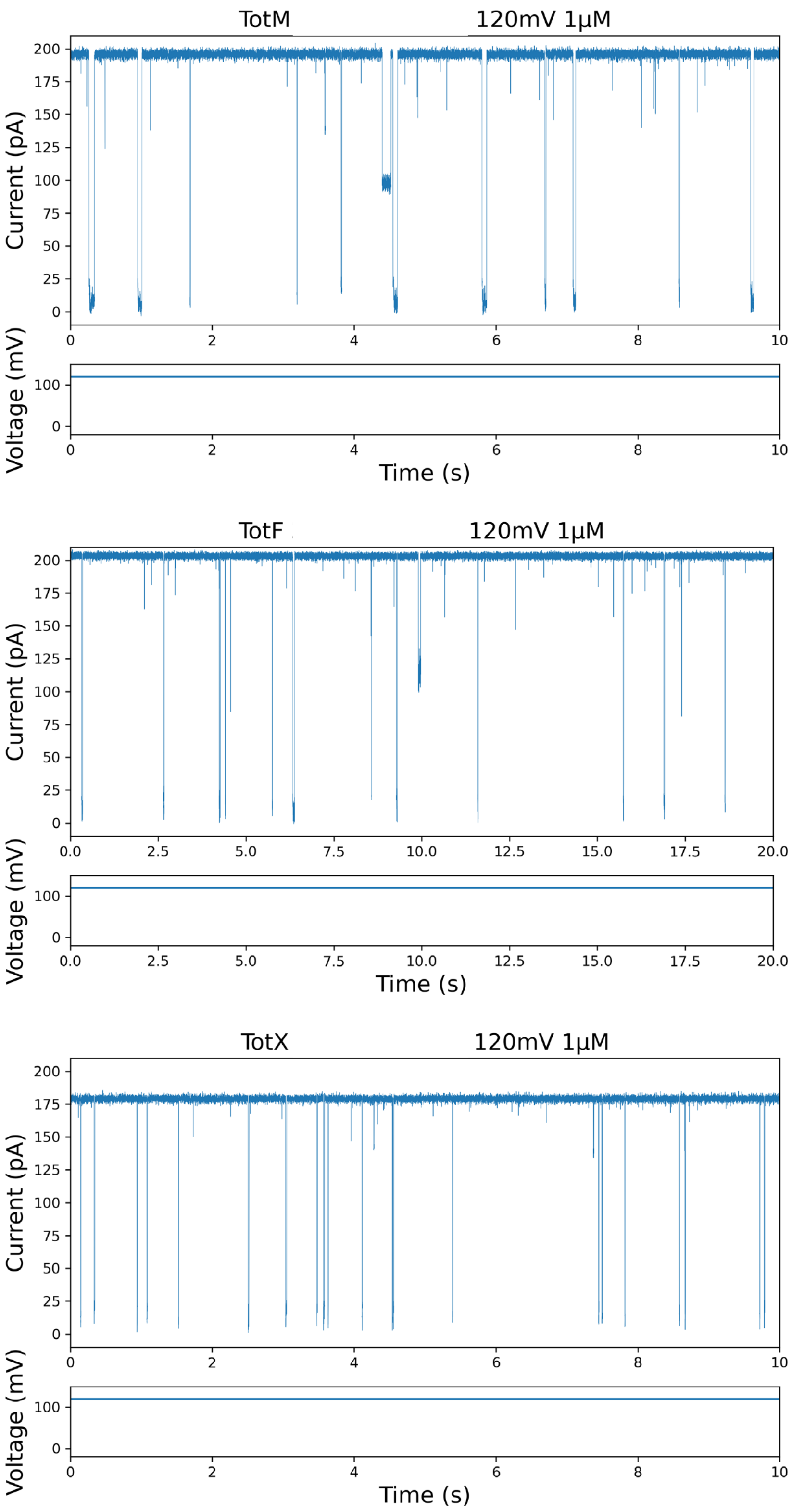

Figure S10. Example traces of TotF, TotM, and TotX recorded in 3 M GdmCl at pH 4.0, 120 mV, filtered with 1 kHz for illustration purpose only.

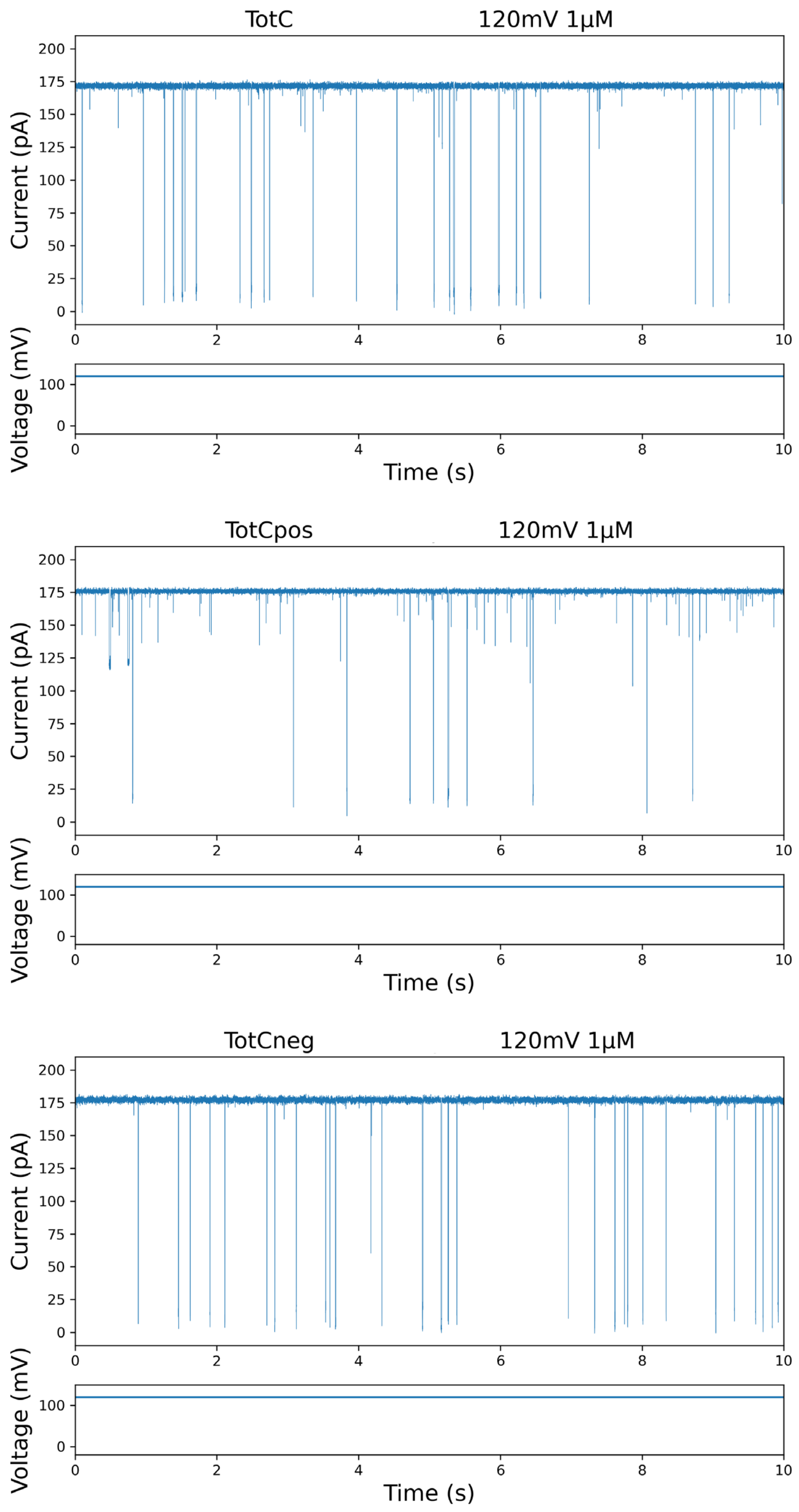

Figure S11. Example traces of TotC, TotCpos, and TotCneg recorded in 3 M GdmCl at pH 4.0, 120 mV, filtered with 1 kHz for illustration purpose only.

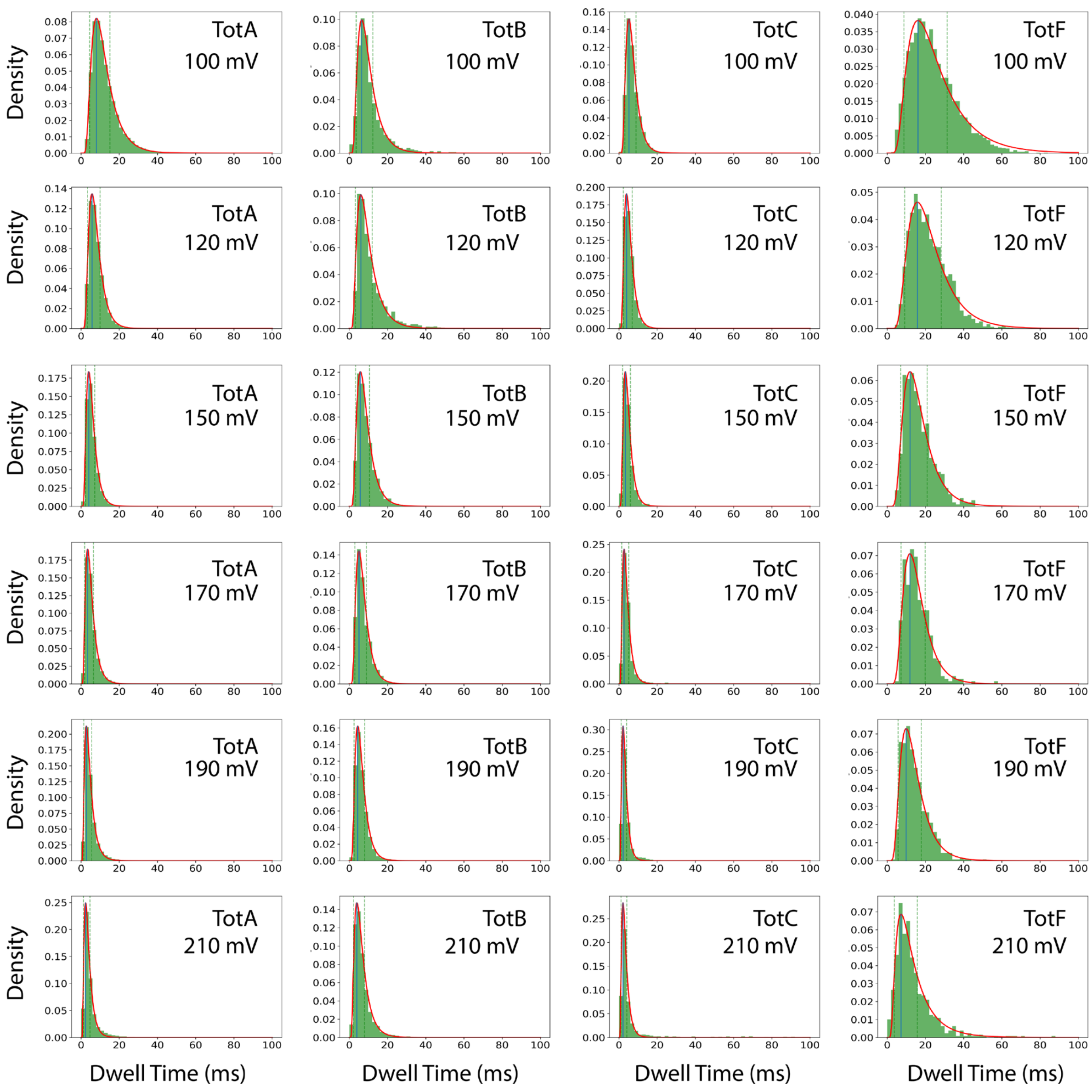

Figure S12. Histograms of dwell times of (columns left to right) TotA, TotB, TotC, and TotF at (rows top to bottom) 100 mV, 120 mV, 150 mV, 170 mV, 190 mV, and 210 mV. In red the fitted Fokker Planck equation is shown with its maximum indicated as a blue line and the distribution half widths as dashed lines.

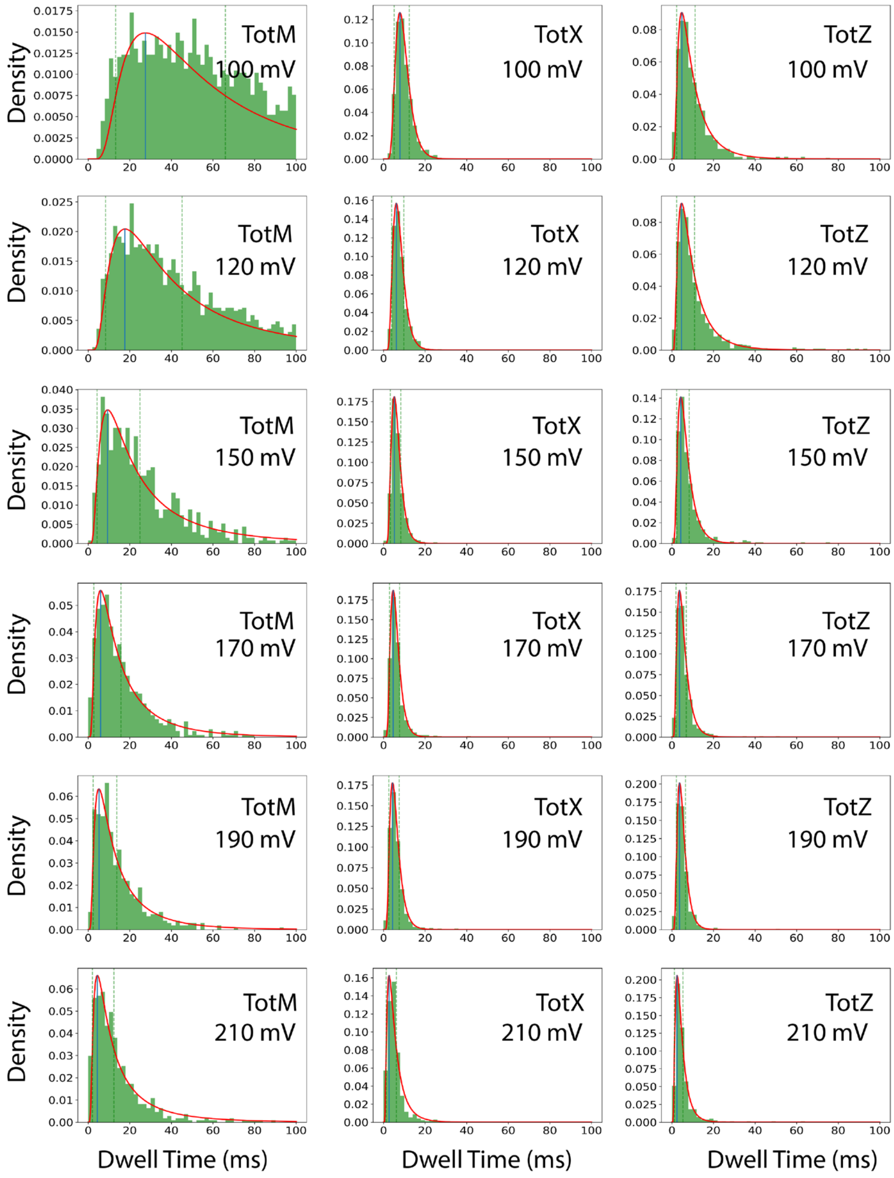

Figure S13. Histograms of dwell times of (columns left to right) TotM, TotX, and TotZ at (rows top to bottom) 100 mV, 120 mV, 150 mV, 170 mV, 190 mV, and 210 mV. In red the fitted Fokker Planck equation is shown with its maximum indicated as a blue line and the distribution half widths as dashed lines.

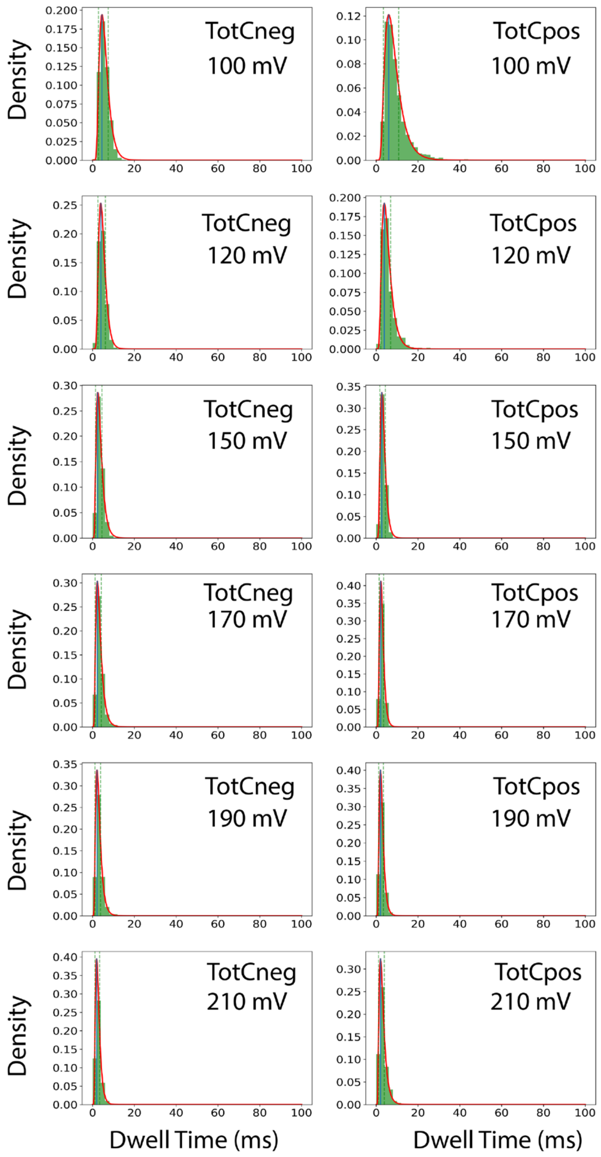

Figure S14. Histograms of dwell times of (columns left to right) TotCneg, and TotCpos at (rows top to bottom) 100 mV, 120 mV, 150 mV, 170 mV, 190 mV, and 210 mV. In red the fitted Fokker Planck equation is shown with its maximum indicated as a blue line and the distribution half widths as dashed lines.

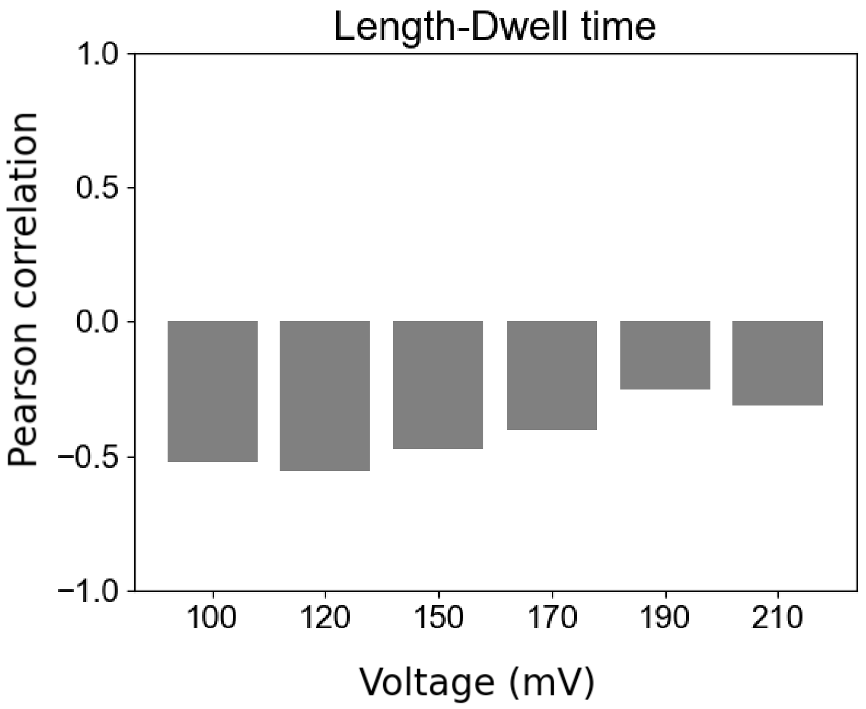

Figure S15. Pearson correlation between analyte length (number of amino acids) and the fitted dwell time for the 7 Tot family proteins across different voltages. P-values in SI Table S6.

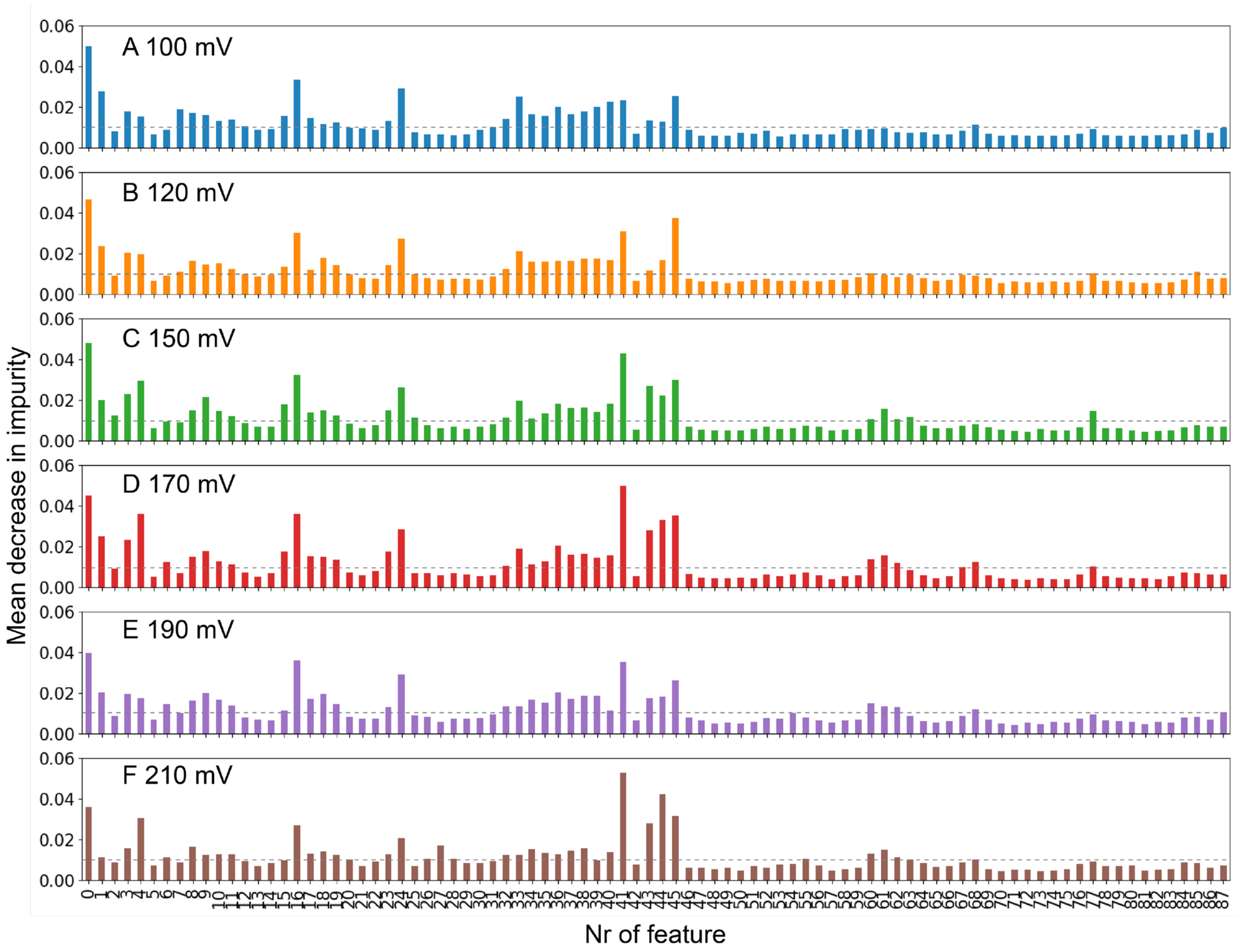

Figure S16. Mean decreases of impurity for 7-way classifications of TotA, TotB, TotC, TotF, TotM, TotX, and TotZ in 3 M GdmCl at pH 4.0 measured with aerolysin K238A at (A) 100 mV, (B) 120 mV, (C) 150 mV, (D) 170 mV, (E) 190 mV, and (F) 210 mV. The feature number corresponds to features listed in SI Table S2. In classifications only features that exceeded the threshold of 0.01 MDI (shown as grey, dashed line) were used.

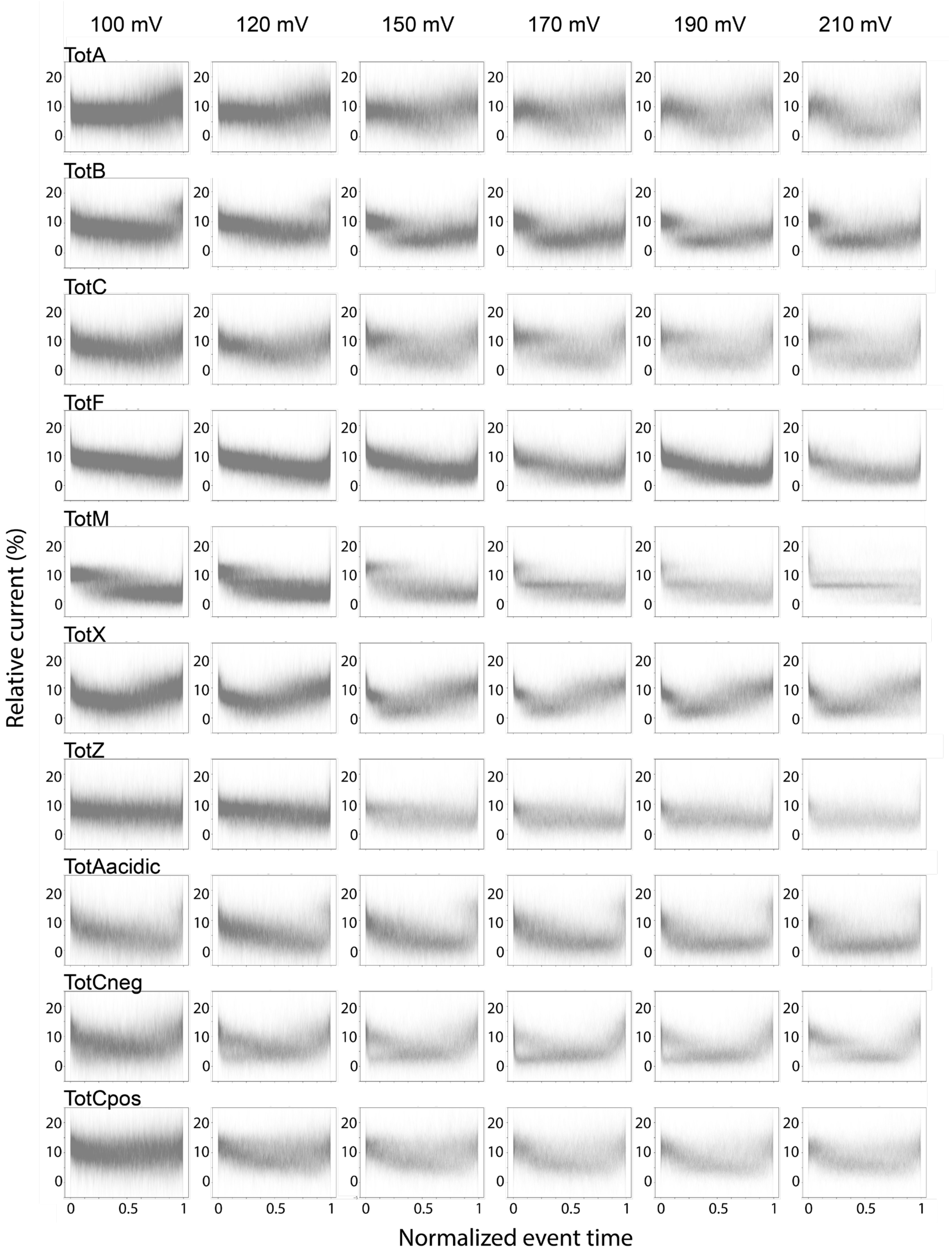

Figure S17. Time normalized overlay plots of randomly chosen events recorded various voltages for each Tot family protein.

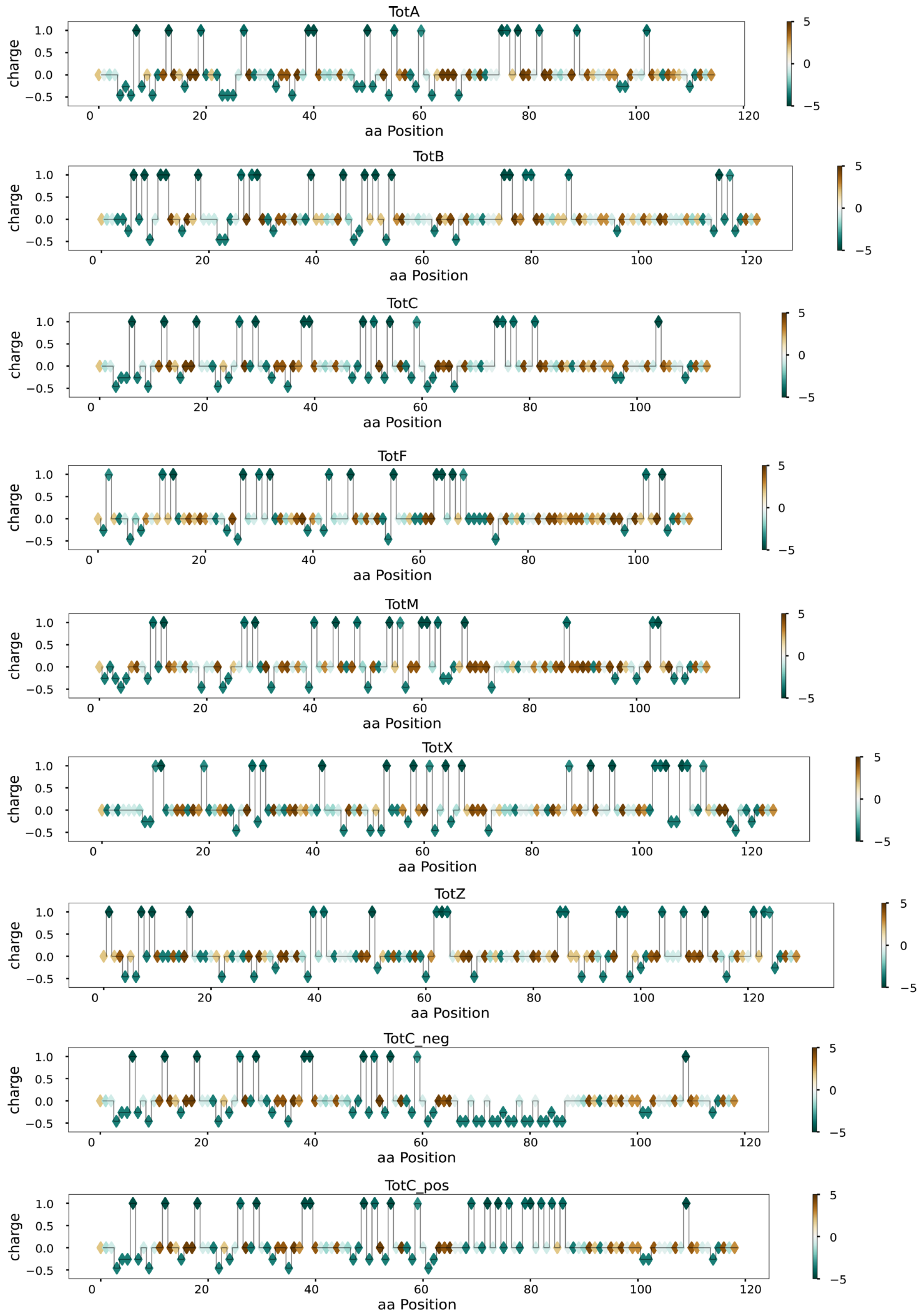

Figure S18. Charge and hydropathy properties along the aa residues of the analyzed proteins.

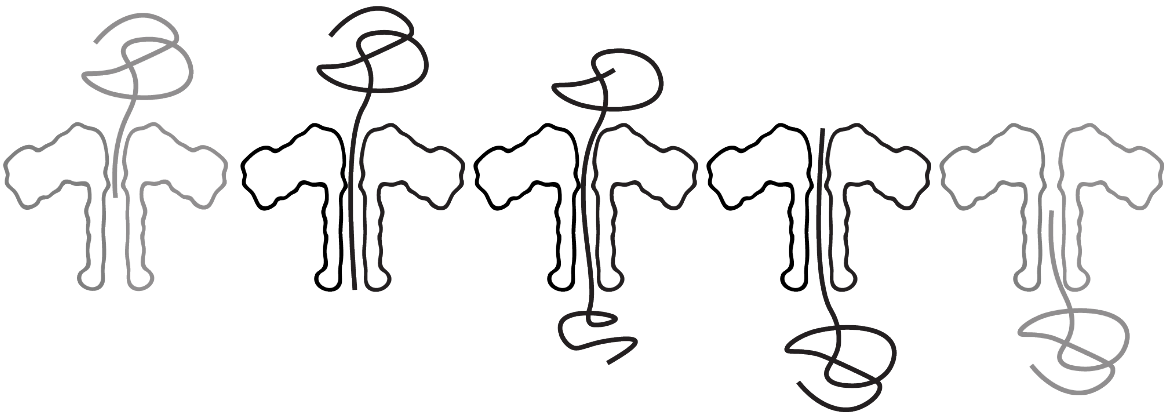

Figure S19. Illustration of the protein translocation scenario in aerolysin nanopore. In the sliding window approach, the beginning and end (gray) of the process are not reflected.

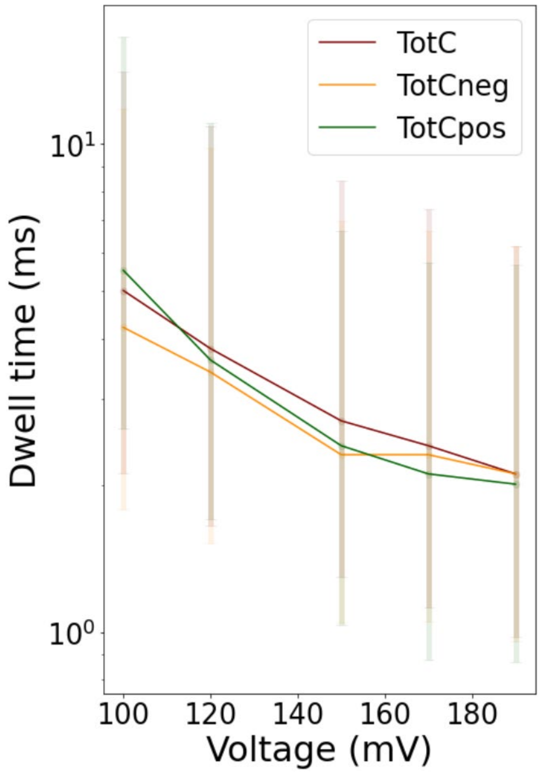

Figure S20. Dwell time trends of TotC, TotCneg and TotCpos. Population dwell times of each proteins were fitted with the Fokker Plank equation and plotted against the applied potential. Maximum and halfwidths of the dwelltime distributions are shown.

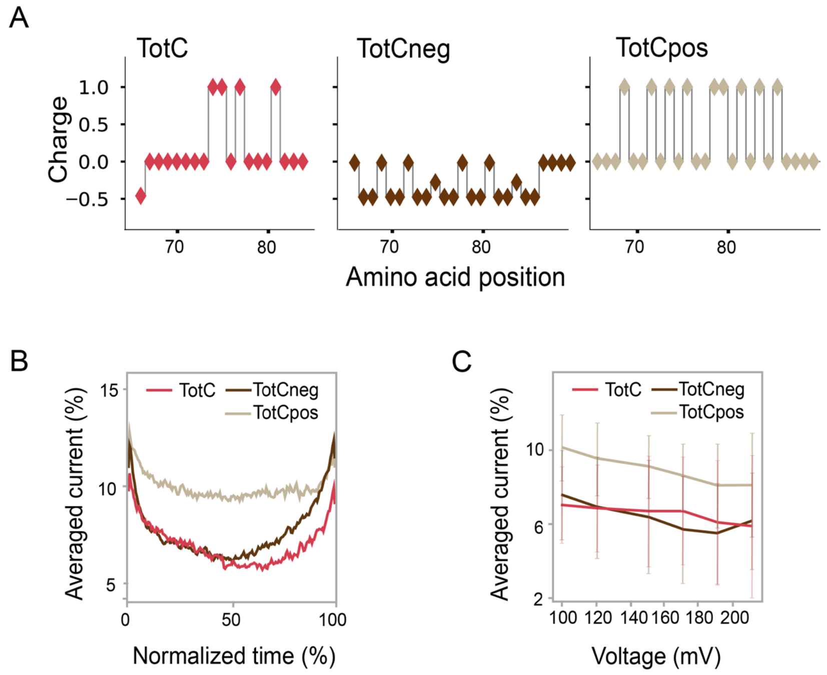

Figure S21. (A) Charge property along the unstructured loop of TotC, TotCneg, and TotCpos (left to right). (B) Average current of 800 randomly chosen events along normalized time recorded at 100 mV for TotC, TotCneg and TotCpos. (C) Population average over the mean currents of TotC (light red), TotCneg (brown), and TotCpos (green).

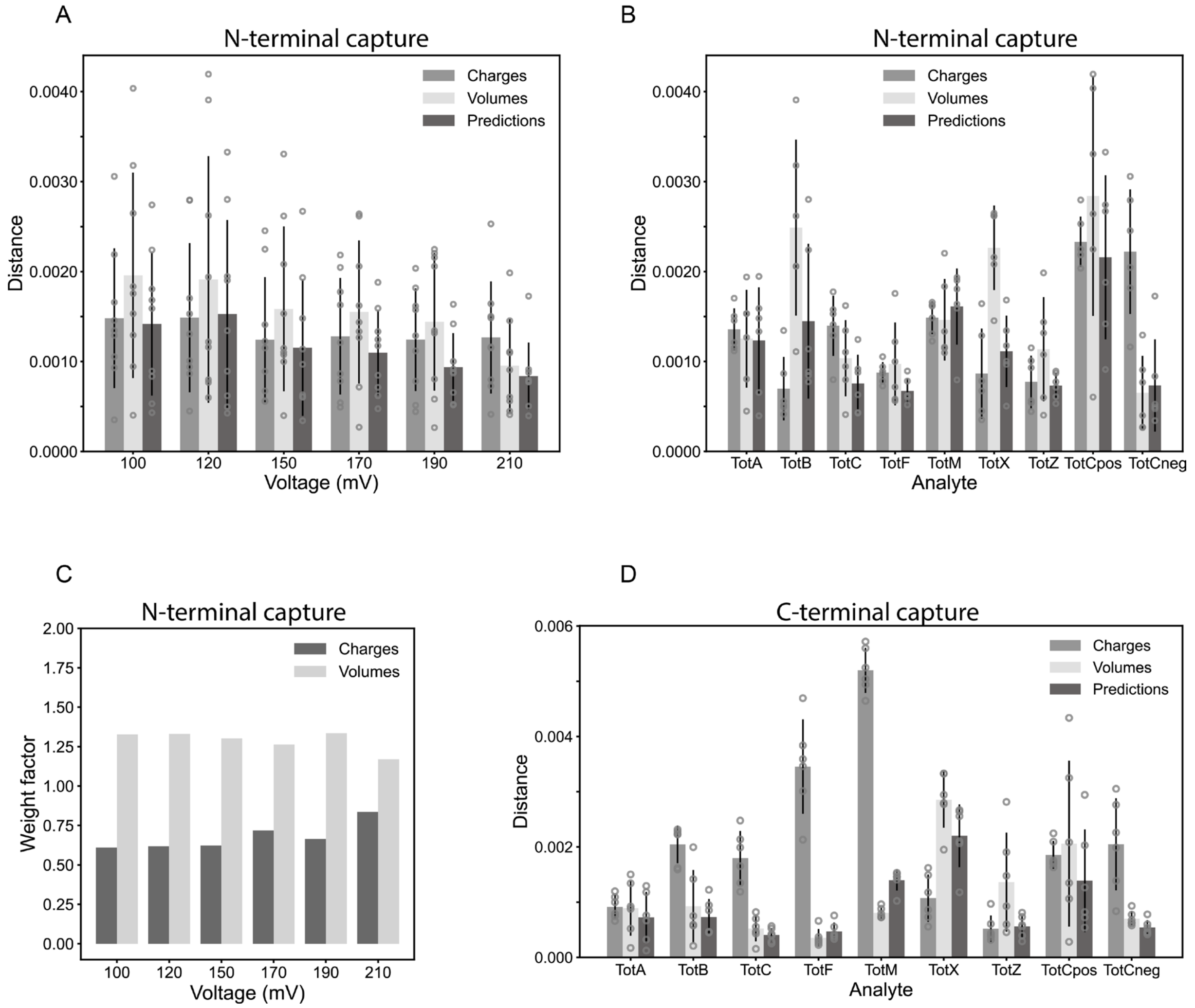

Figure S22. Additional signal pattern analysis. (A) Weighted DTW distances between the consensuses and positive charges (dark grey), residual volumes (light grey), and the prediction (black) shown at each voltage when assuming N-terminal capture. Bars represent the average with values from individual proteins shown as dots. (B) Weight factors for positive charges (dark grey) and residual volumes (light grey) optimized for the predictions for each voltage when assuming N-terminal capture. (C) Weighted DTW distances that were shown in (A) but displayed as an average over voltages for each analyte when assuming N-terminal capture. (D) Weighted DTW distances corresponding to Fig 5E but displayed as average over voltages for each analyte. Shown for C-terminal capture.

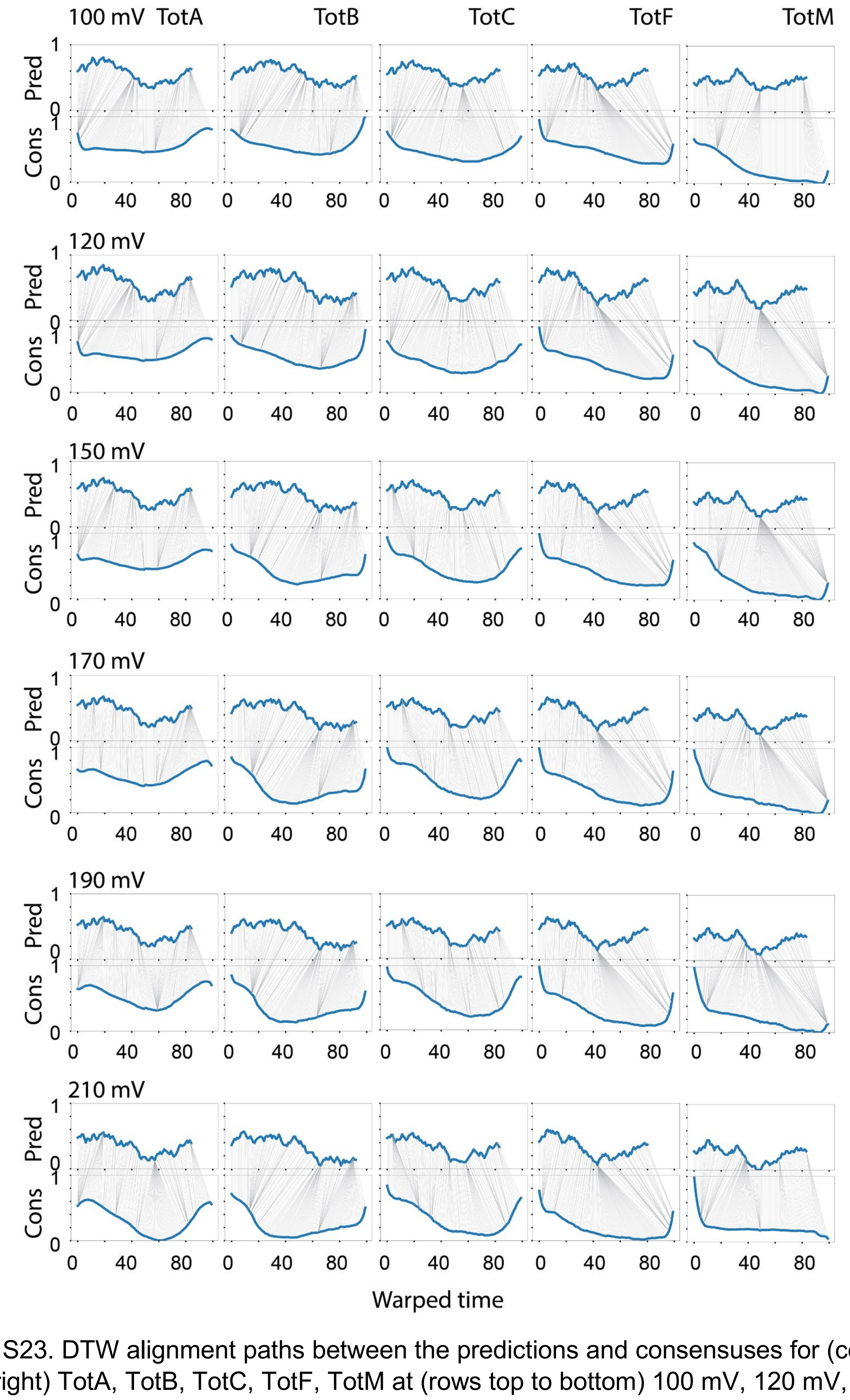

Figure S23. DTW alignment paths between the predictions and consensuses for (columns left to right) TotA, TotB, TotC, TotF, TotM at (rows top to bottom) 100 mV, 120 mV, 150 mV, 170 mV, 190 mV, and 210 mV. Shown for C-terminal capture analysis.

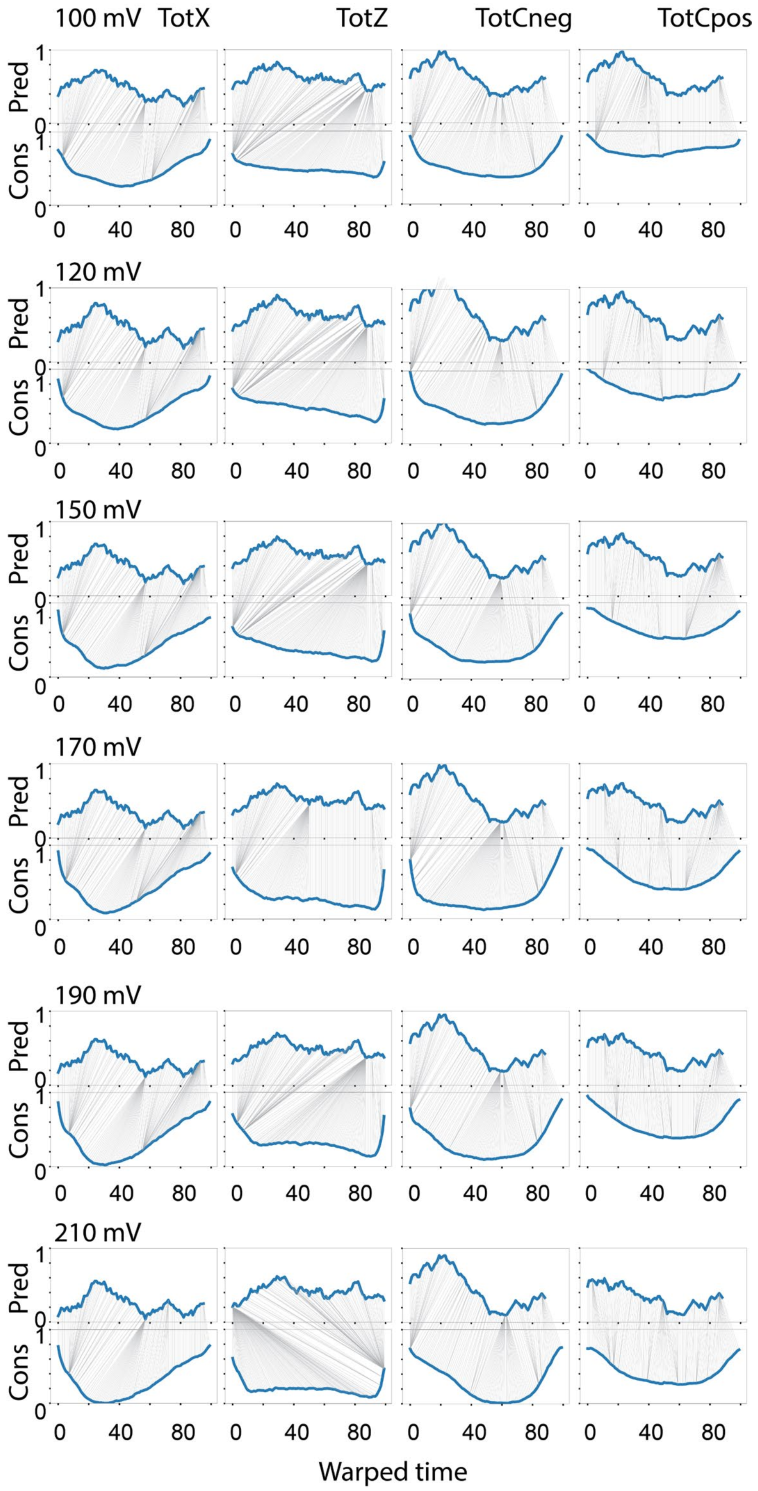

Figure S24. DTW alignment paths between the predictions and consensuses for (columns left to right) TotX, TotZ, TotCneg, TotCpos at (rows top to bottom) 100 mV, 120 mV, 150 mV, 170 mV, 190 mV, and 210 mV. Shown for C-terminal capture.

References

1. Cao, C. *et al.* Deep Learning-Assisted Single-Molecule Detection of Protein Post-translational Modifications with a Biological Nanopore. *ACS Nano* **18**, 1504–1515 (2024).
2. Rommelaere, S. *et al.* A humoral stress response protects Drosophila tissues from antimicrobial peptides. *Curr. Biol.* **34**, 1426-1437.e6 (2024).
3. Macaskill, J. B., Robinson, R. A. & Bates, R. G. Osmotic coefficients and activity coefficients of guanidinium chloride in concentrated aqueous solutions at 25.degree.C. *J. Chem. Eng. Data* **22**, 411–412 (1977).
4. CRC Handbook of Chemistry and Physics, 84th Edition Edited by David R. Lide (National Institute of Standards and Technology). CRC Press LLC: Boca Raton. 2003. 2616 pp. $139.95. ISBN 0-8493-0484-9. *J. Am. Chem. Soc.* **126**, 1586–1586 (2004).
5. Hille, B. *Ion Channels of Excitable Membranes*. *Ion Channels of Excitable Membranes* vol. 18 814 (2001).
6. Micsonai, A. *et al.* Accurate secondary structure prediction and fold recognition for circular dichroism spectroscopy. *Proc. Natl. Acad. Sci.* **112**, E3095–E3103 (2015).
7. Ling, D. Y. & Ling, X. S. On the distribution of DNA translocation times in solid-state nanopores: an analysis using Schrödinger's first-passage-time theory. *J. Phys. Condens. Matter Inst. Phys. J.* **25**, 375102 (2013).
8. Cao, C. *et al.* Aerolysin nanopores decode digital information stored in tailored macromolecular analytes. *Sci. Adv.* **6**, eabc2661 (2021).
9. Im, J., Lindsay, S., Wang, X. & Zhang, P. Single Molecule Identification and Quantification of Glycosaminoglycans Using Solid-State Nanopores. *ACS Nano* **13**, 6308–6318 (2019).
10. Anton, J. S. *et al.* Aerolysin nanopore structure revealed at high resolution in lipid environment. 2024.08.12.607338 Preprint at https://doi.org/10.1101/2024.08.12.607338 (2024).
11. Jo, S., Kim, T., Iyer, V. G. & Im, W. CHARMM-GUI: a web-based graphical user interface for CHARMM. *J. Comput. Chem.* **29**, 1859–1865 (2008).

12. Huang, J. & MacKerell, A. D. CHARMM36 all-atom additive protein force field: validation based on comparison to NMR data. *J. Comput. Chem.* **34**, 2135–2145 (2013).

13. Ploetz, E. A. *et al.* Kirkwood-Buff-Derived Force Field for Peptides and Proteins: Philosophy and Development of KBFF20. *J. Chem. Theory Comput.* **17**, 2964–2990 (2021).

14. Lomize, M. A., Pogozheva, I. D., Joo, H., Mosberg, H. I. & Lomize, A. L. OPM database and PPM web server: resources for positioning of proteins in membranes. *Nucleic Acids Res.* **40**, D370–D376 (2012).

15. Van Der Spoel, D. *et al.* GROMACS: Fast, flexible, and free. *J. Comput. Chem.* **26**, 1701–1718 (2005).

16. Case, D. A. *et al.* The Amber biomolecular simulation programs. *J. Comput. Chem.* **26**, 1668–1688 (2005).

17. Jurrus, E. *et al.* Improvements to the APBS biomolecular solvation software suite. *Protein Sci. Publ. Protein Soc.* **27**, 112–128 (2018).

18. Darden, T., York, D. & Pedersen, L. Particle mesh Ewald: An N·log(N) method for Ewald sums in large systems. *J. Chem. Phys.* **98**, 10089–10092 (1993).

19. Smart, O. S., Neduvelil, J. G., Wang, X., Wallace, B. A. & Sansom, M. S. HOLE: a program for the analysis of the pore dimensions of ion channel structural models. *J. Mol. Graph.* **14**, 354–60, 376 (1996).

20. Mehrafrooz, B. *et al.* Electro-osmotic Flow Generation via a Sticky Ion Action. *ACS Nano* **18**, 17521–17533 (2024).

21. Perkins, S. J. Protein volumes and hydration effects. *Eur. J. Biochem.* **157**, 169–180 (1986).

22. Halligan, B. D. ProMoST: A tool for calculating the pI and molecular mass of phosphorylated and modified proteins on 2 dimensional gels. *Methods Mol. Biol. Clifton NJ* **527**, 283–ix (2009).

23. Kyte, J. & Doolittle, R. F. A simple method for displaying the hydropathic character of a protein. *J. Mol. Biol.* **157**, 105–132 (1982).

24. Jeong, Y.-S., Jeong, M. K. & Omitaomu, O. A. Weighted dynamic time warping for time series classification. *Pattern Recognit.* **44**, 2231–2240 (2011).

25. Boukhet, M. *et al.* Probing driving forces in aerolysin and α-hemolysin biological nanopores: electrophoresis versus electroosmosis. *Nanoscale* **8**, 18352–18359 (2016).

26. Protein Tool. https://www.protpi.ch/Calculator/ProteinTool.
